# Supplementary figures and images for: The actions of others act as a pseudo-reward to drive imitation in the context of social reinforcement learning
Source: PLoS Biol. 2020 Dec 8;18(12):e3001028. doi: 10.1371/journal.pbio.3001028 (PMC7723279; doi:10.1371/journal.pbio.3001028)

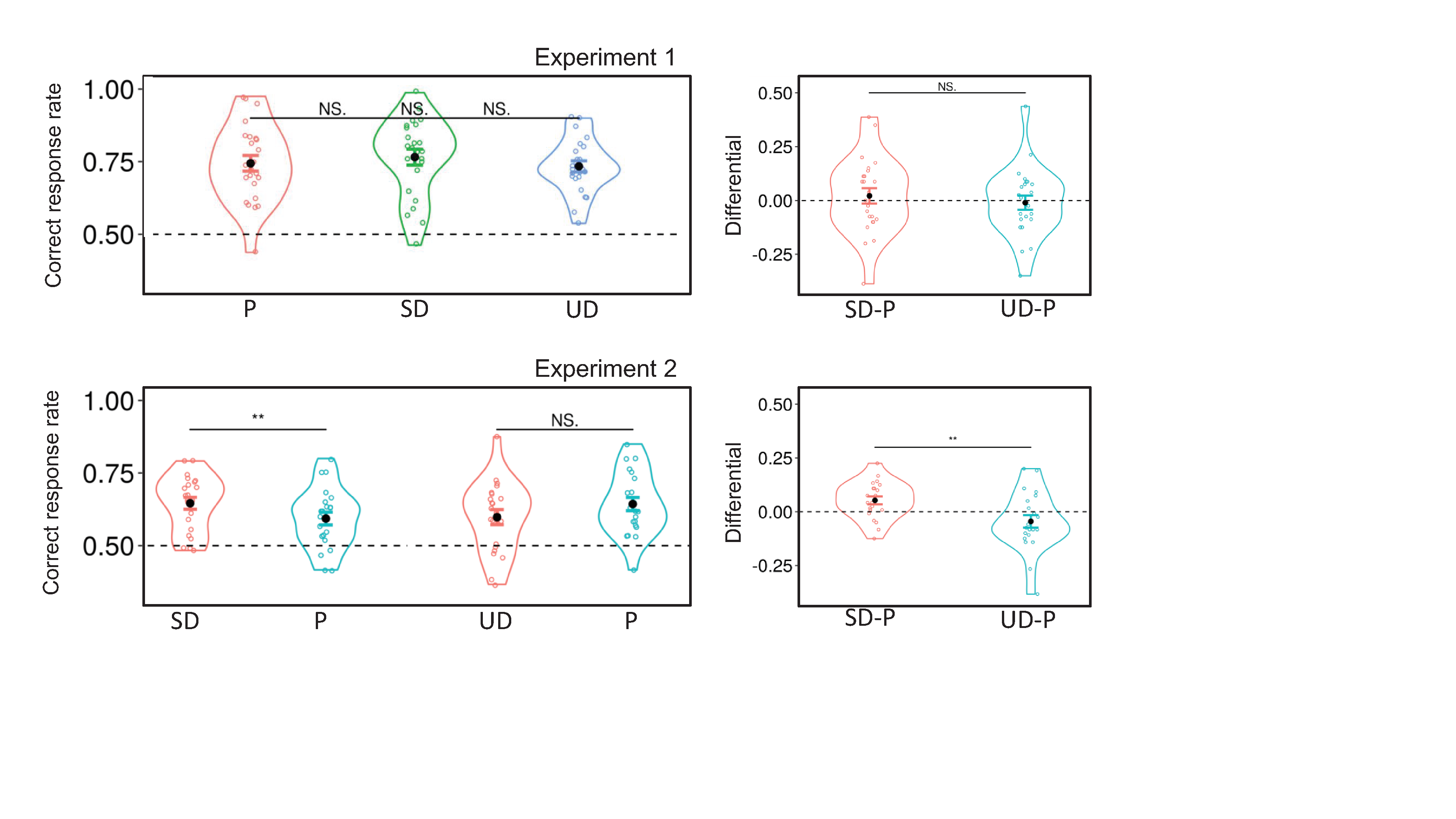

Supplement: S1 Fig — P private condition. SD and UD observational conditions. In Exp 1, task performance in the SD condition was higher than in the Private condition (P), which itself was higher than performance in the UD condition. However, this difference in performance was not statistically significant. In Exp 2, the differential in task performance between observational and private conditions was statistically different between the SD and the UD group. Underlying data can be found in https://github.com/hrl-team/mfree_imitation/. SD, Skilled Demonstrator; UD, Unskilled Demonstrator. (TIF) [file pbio.3001028.s001.tif]

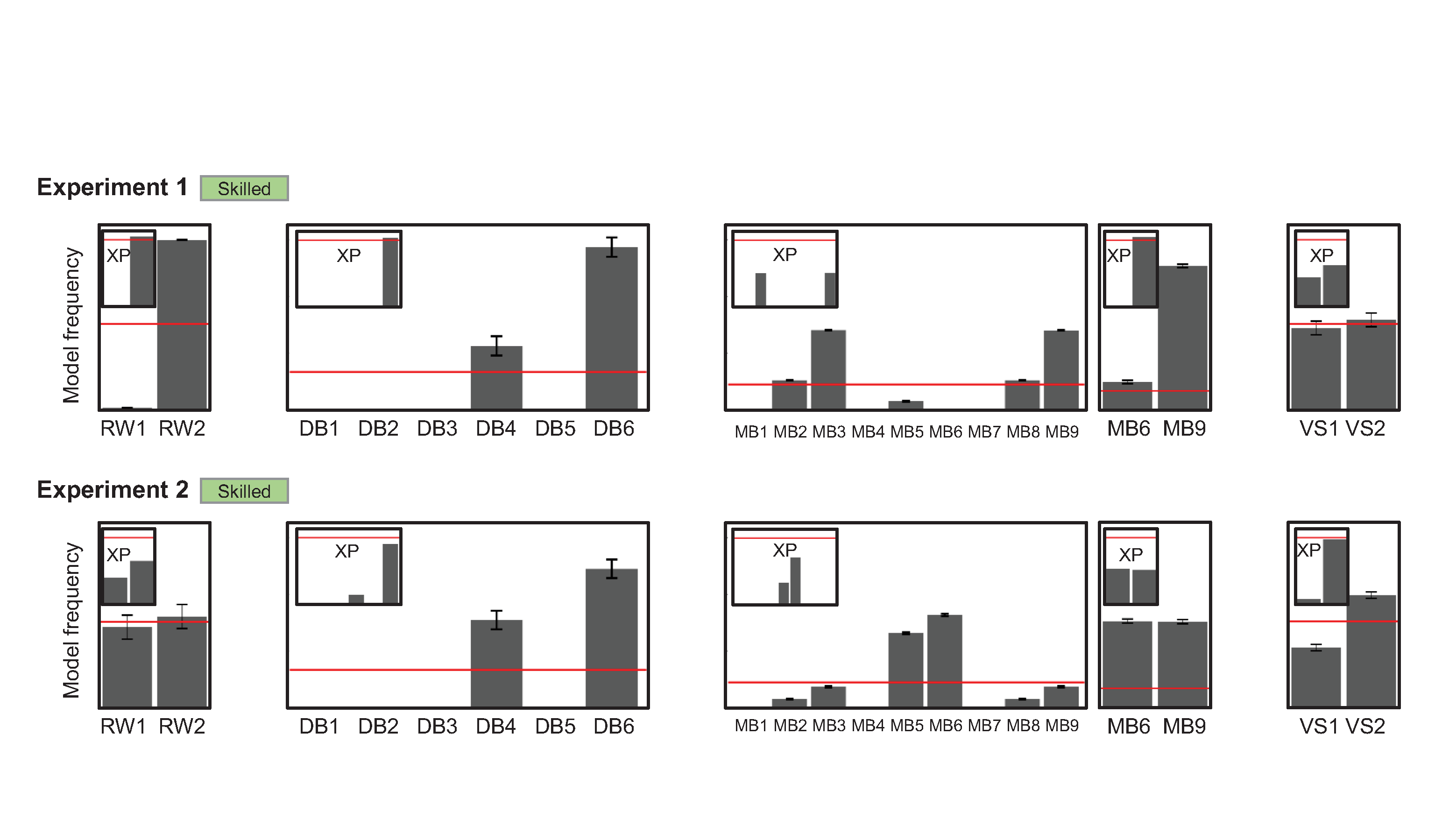

Supplement: S2 Fig — We compared different implementations of each model: 2 for RW, 6 for DB, 9 for MB, and 2 for VS. For the baseline, implementing a symmetric value update (RW2) improves the model fitting quality in Exp 1. For DB, the best fitting performance is achieved when allowing for symmetric value update from observed demonstrations and for the accumulation of successive demonstrations (DB6). For MB, the best implementations use the model of the Demonstrator for biasing Learner’s actions through symmetric value update (MB3 and MB9 in Exp 1 and MB6 in Exp 2). A finer comparison between MB3 and MB9 in both experiments shows that these models are equivalent (not shown here). A further comparison between MB6 and MB9 in both experiments shows that MB9 fits better than MB6. Finally, the best VS implementation, VS2, uses a symmetric value update from observed demonstrations. As a result of this analysis, the final model space includes RW2, DB6, MB9, and VS2. Underlying data can be found in https://github.com/hrl-team/mfree_imitation/. DB, decision biasing; MB, model-based imitation; RW, Rescorla–Wagner; VS, value shaping. (TIF) [file pbio.3001028.s002.tif]

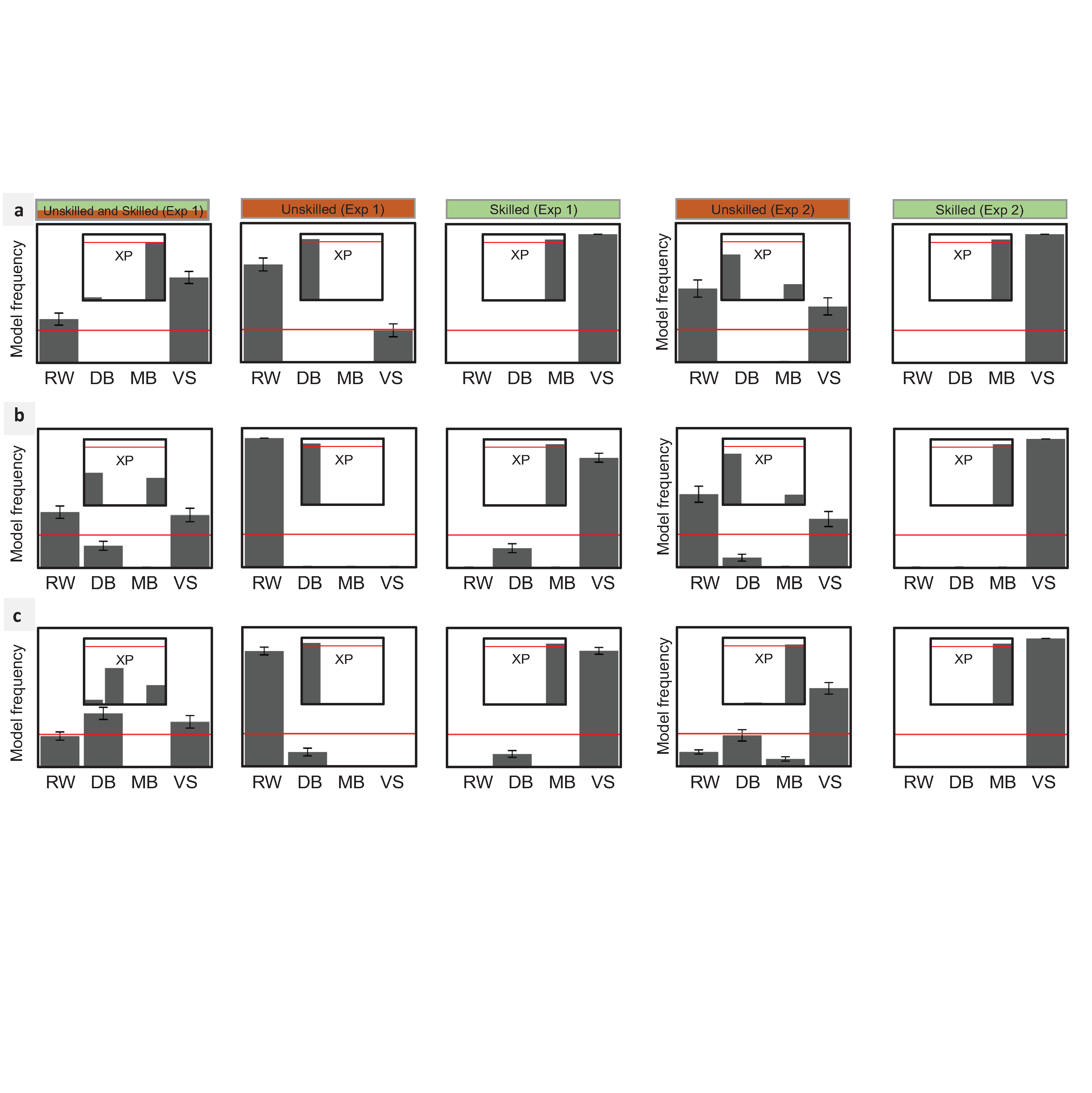

Supplement: S3 Fig — (a) Model space implementation without choice autocorrelation parameter. (b) Model space implementation without symmetric value update for private learning. (c) Model space implementation allowing for negative imitation learning rates. Note that in Exp 2, when allowing for negative learning rates, the winning model in the UD condition is no longer RW, but VS. Underlying data can be found in https://github.com/hrl-team/mfree_imitation/. RW, Rescorla–Wagner; UD, Unskilled Demonstrator; VS, value shaping. (TIF) [file pbio.3001028.s003.tif]

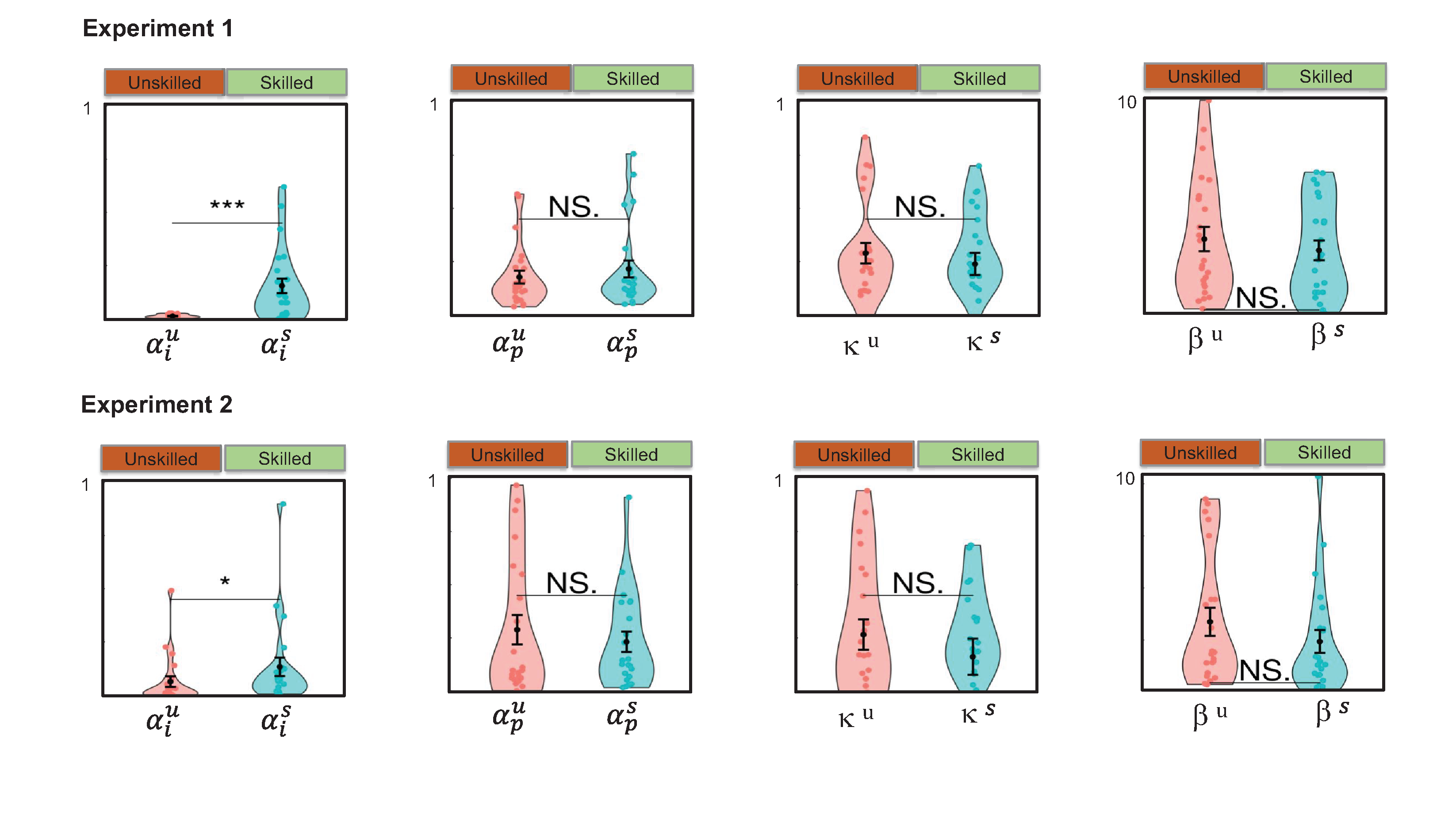

Supplement: S4 Fig — No statistical difference was found for nonsocial parameters αp, κ, and β. Only the imitation learning rate αi was statistically different across observational conditions in both experiments. Underlying data can be found in https://github.com/hrl-team/mfree_imitation/. SD, Skilled Demonstrator; UD, Unskilled Demonstrator. (TIF) [file pbio.3001028.s004.tif]

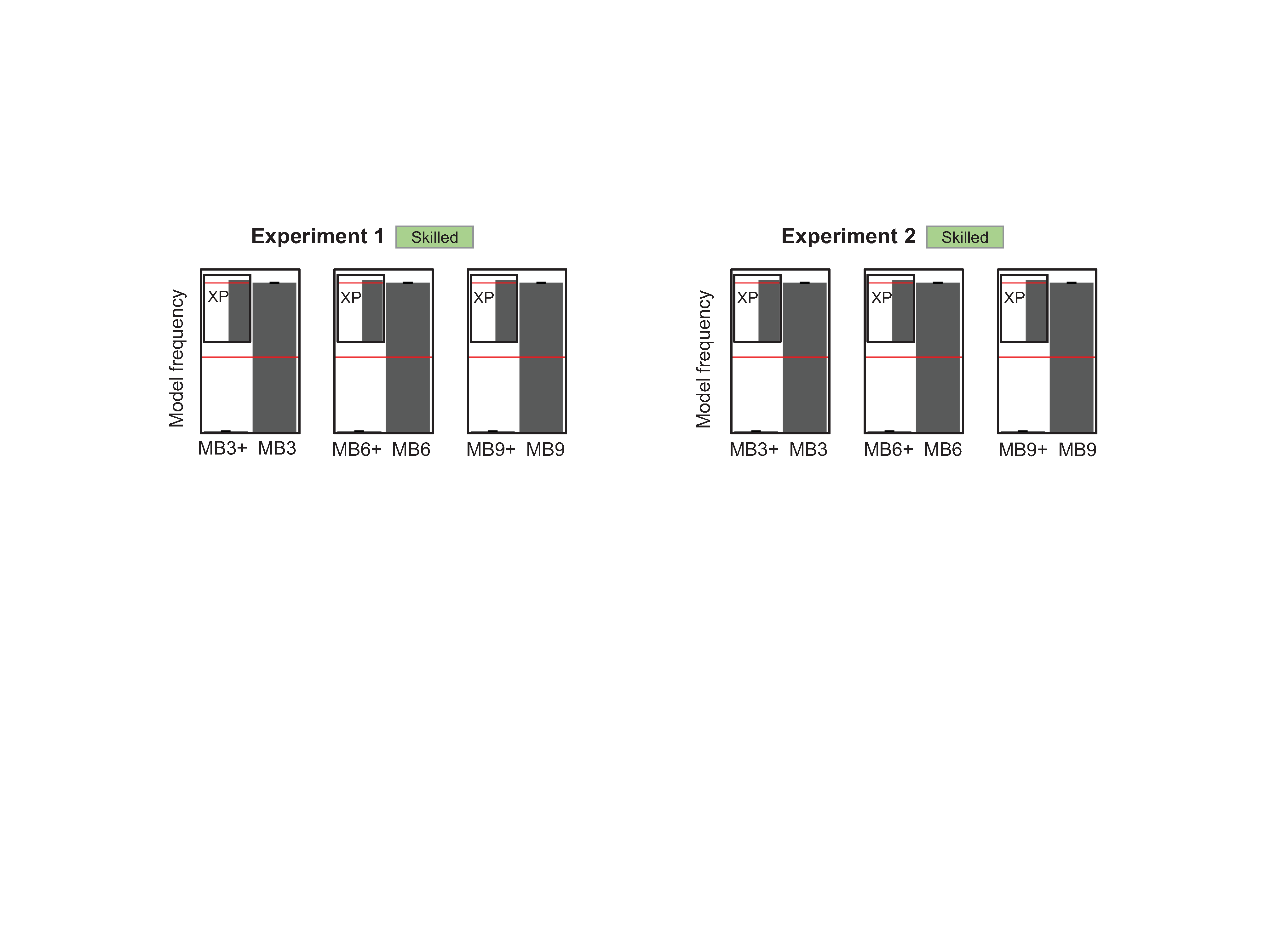

Supplement: S5 Fig — Specifically, implementations with a fixed parameter αd = 0.1 (MB3, MB6, and MB9) are better than implementations with αd as a free parameter (MB3+, MB6+, and MB9+, respectively). αd is the learning rate used for inferring the preferences of the Demonstrator. Underlying data can be found in https://github.com/hrl-team/mfree_imitation/. MB, model-based imitation. (TIF) [file pbio.3001028.s005.tif]

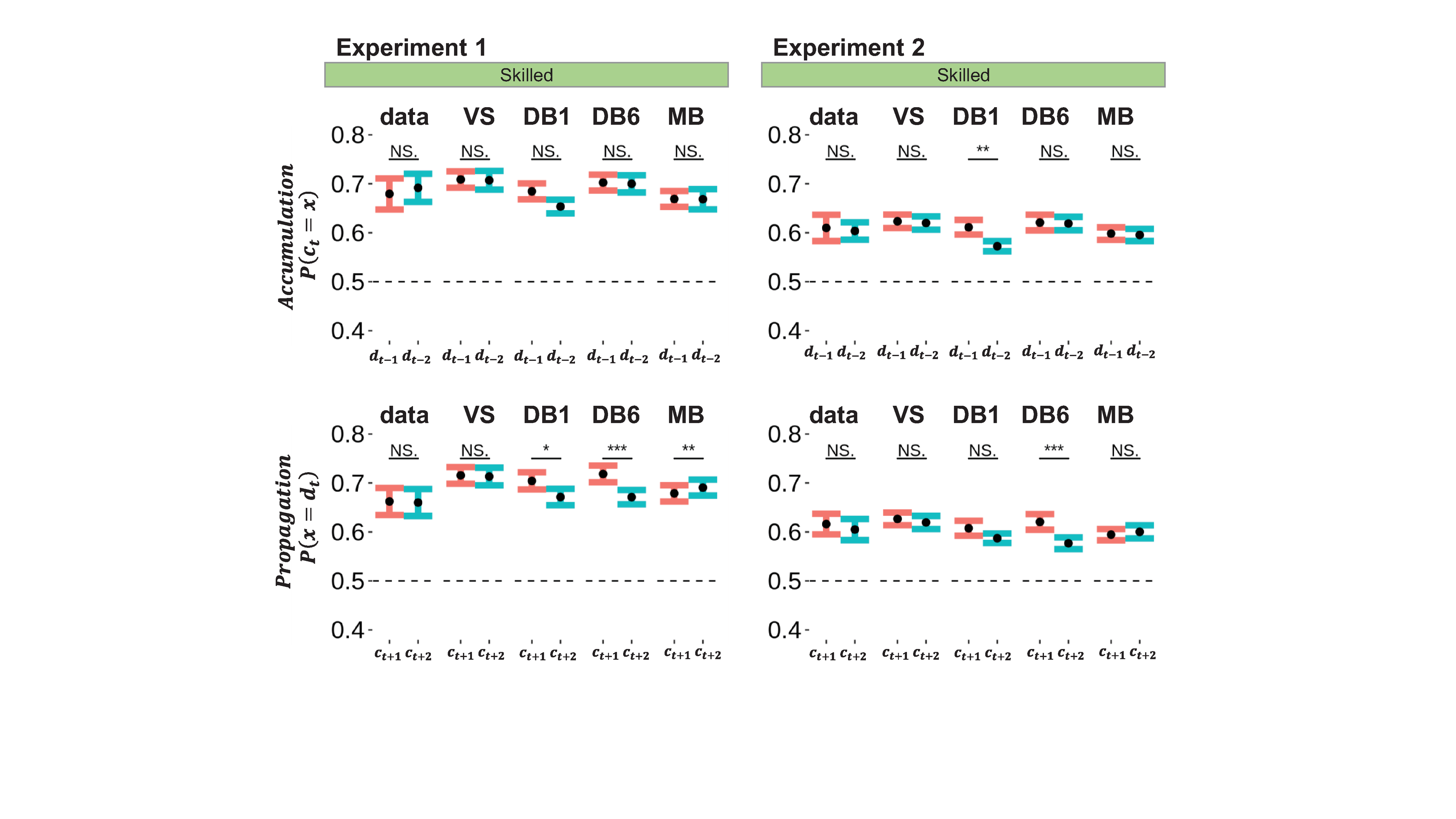

Supplement: S6 Fig — Accumulation (top row): behavioral imitation rate calculated as a function of the 2 preceding demonstrations (dt−1, dt−2). Propagation (bottom row): behavioral imitation rate calculated as a function of 2 consecutive choices (ct+1, ct+2). *p<0.05, **p<0.01, ***p<0.001, paired t test. Underlying data can be found in https://github.com/hrl-team/mfree_imitation/. (TIF) [file pbio.3001028.s006.tif]

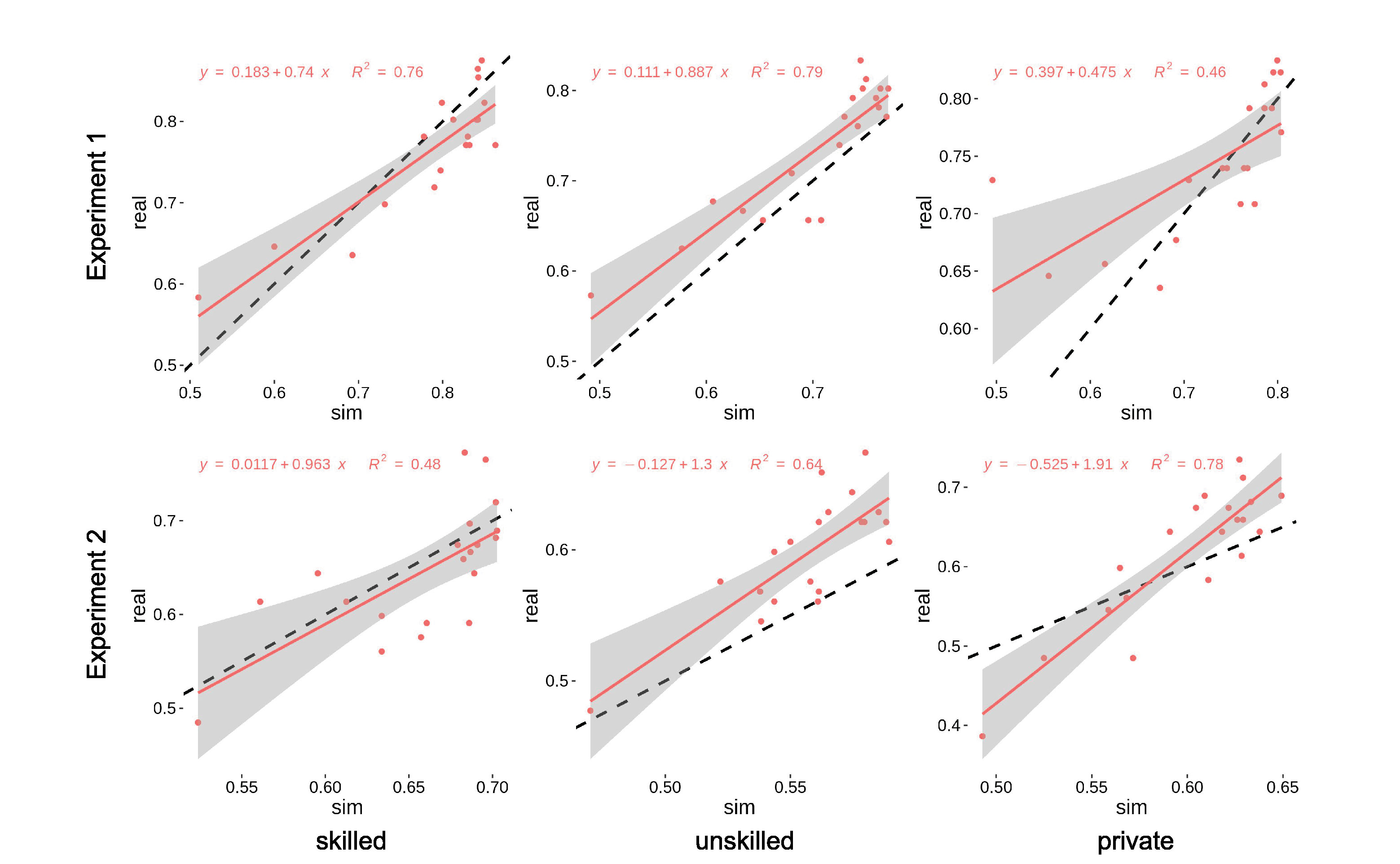

Supplement: S7 Fig — Across-trial correlation between the observed and model-predicted choices. Results are given as Spearman’s correlations. Underlying data can be found in https://github.com/hrl-team/mfree_imitation/. (TIF) [file pbio.3001028.s007.tif]

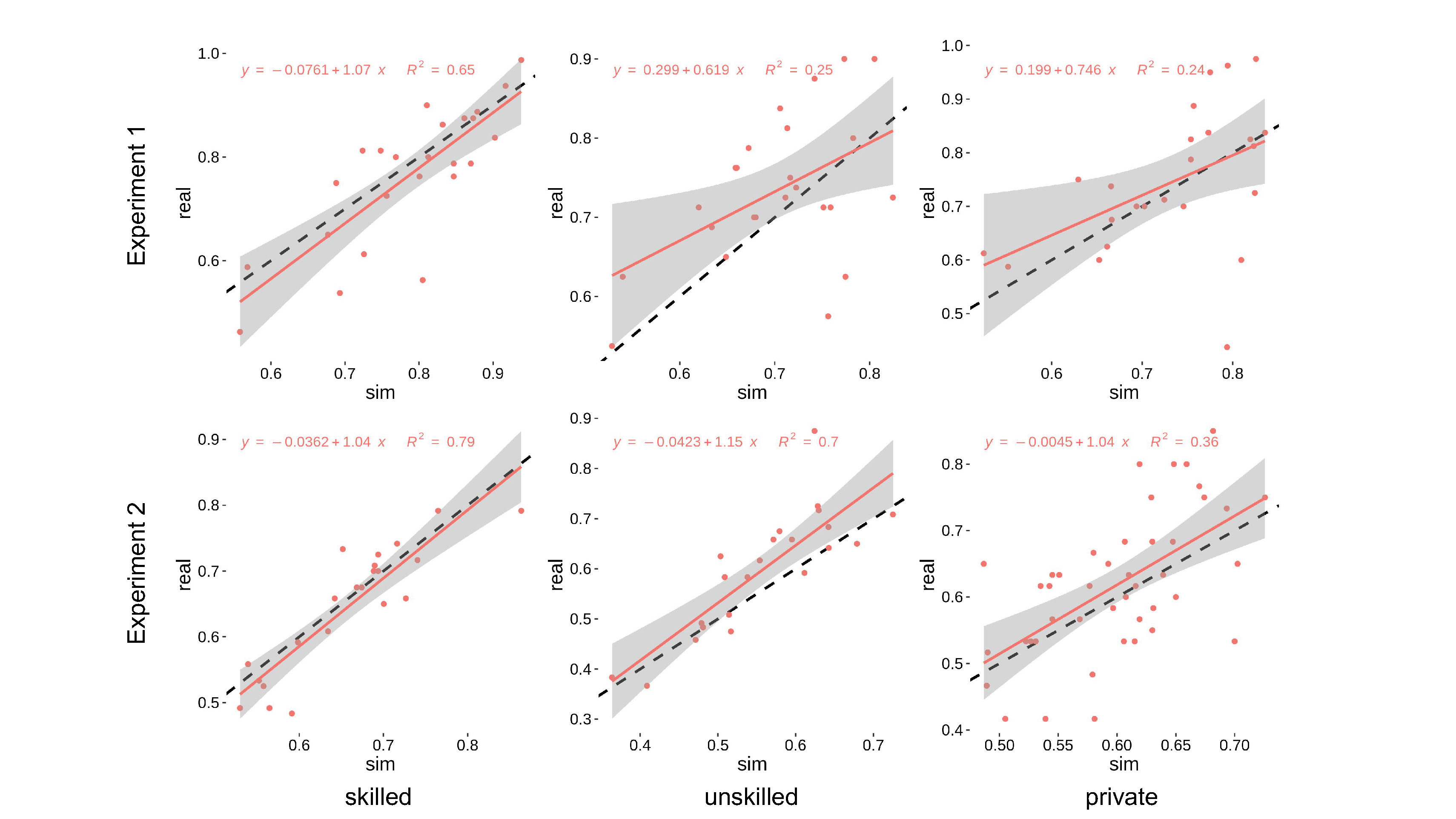

Supplement: S8 Fig — Across-subject correlation between the observed and model-predicted choices. Results are given as Spearman’s correlations. Underlying data can be found in https://github.com/hrl-team/mfree_imitation/. (TIF) [file pbio.3001028.s008.tif]

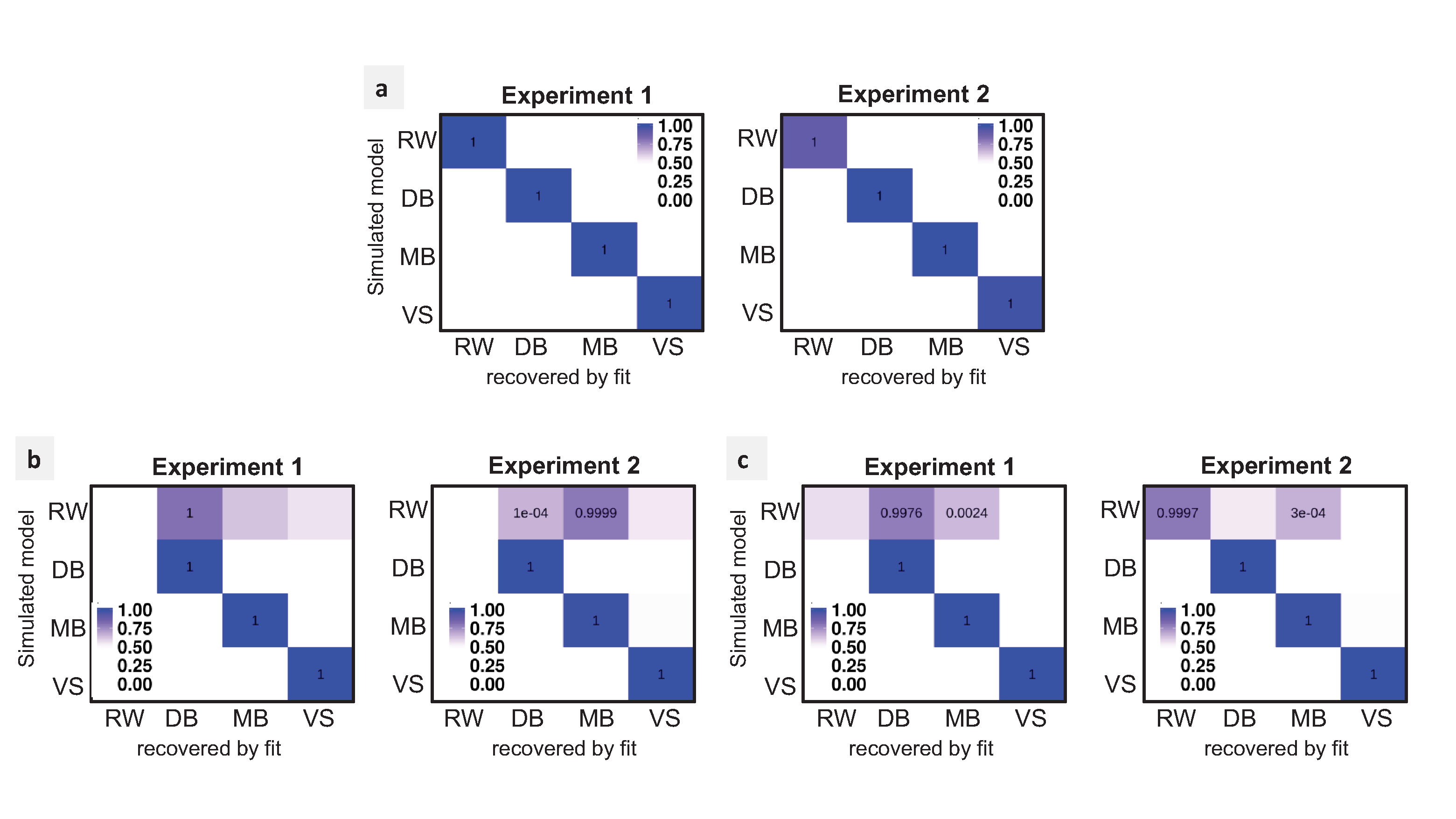

Supplement: S9 Fig — We tested different approximations of the model evidence that we fed to the Variational Bayesian Analysis toolbox. (a) AIC. (b) log-likelihood. (c) log posterior probability. Only AIC displayed a good parameter recovery. AIC, Akaike Information Criterion. (TIF) [file pbio.3001028.s009.tif]

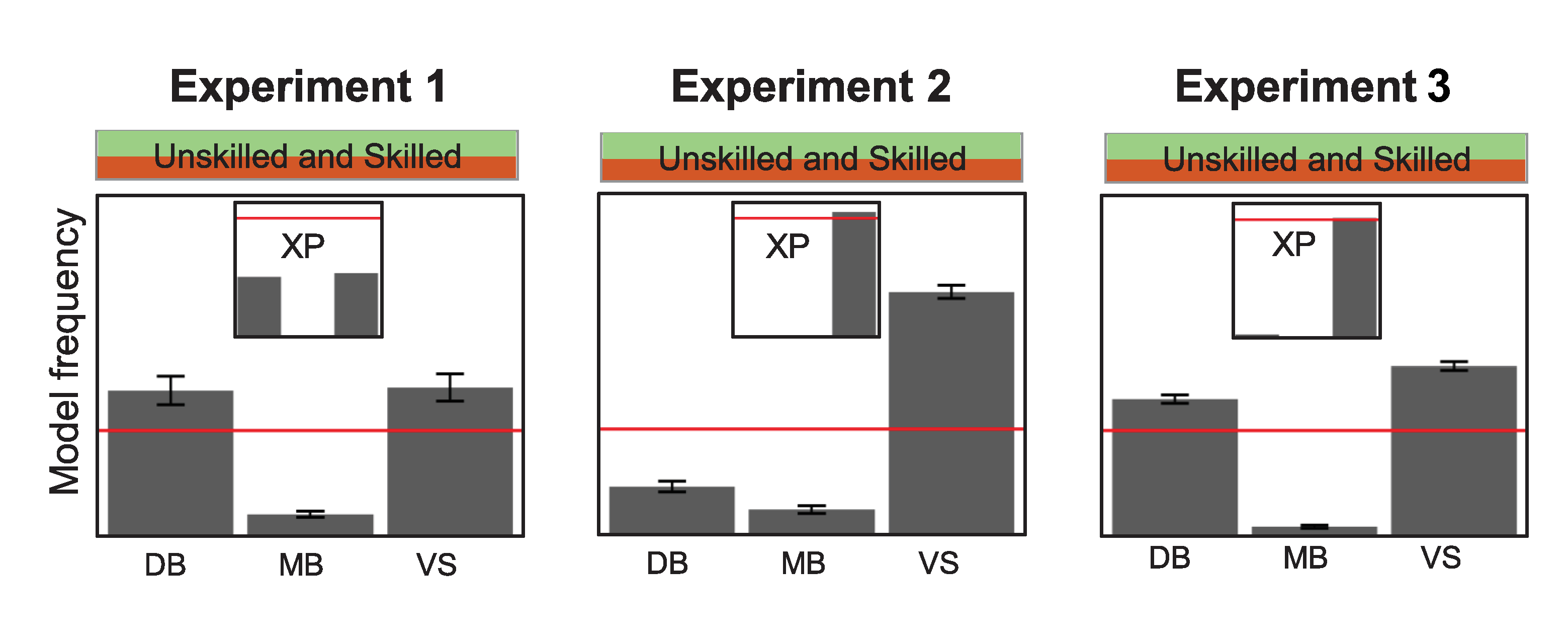

Supplement: S10 Fig — We compared the main imitation models (DB, MB, and VS) with maximum likelihood because they present the same number of free parameters. Underlying data can be found in https://github.com/hrl-team/mfree_imitation/. (TIF) [file pbio.3001028.s010.tif]
