# Supplementary material for: The actions of others act as a pseudo-reward to drive imitation in the context of social reinforcement learning
Source: PLoS Biol. 2020 Dec 8;18(12):e3001028. doi: 10.1371/journal.pbio.3001028 (PMC7723279; doi:10.1371/journal.pbio.3001028)
Supplement: Attachment — Submitted filename: Najar_PlosBio_Rebuttal.pdf [file pbio.3001028.s011.pdf]

Request:

> However, before we can send your manuscript to reviewers, we need you to complete your submission by providing the metadata that is required for full assessment. To this end, please login to Editorial Manager where you will find the paper in the 'Submissions Needing Revisions' folder on your homepage. Please click 'Revise Submission' from the Action Links and complete all additional questions in the submission questionnaire.

Response:
Additional meta-data have been provided.
