# Supplementary material for: The actions of others act as a pseudo-reward to drive imitation in the context of social reinforcement learning
Source: PLoS Biol. 2020 Dec 8;18(12):e3001028. doi: 10.1371/journal.pbio.3001028 (PMC7723279; doi:10.1371/journal.pbio.3001028)
Supplement: Attachment — Submitted filename: Najar_PlosBio_Rebuttal.pdf [file pbio.3001028.s012.pdf]

Reviewer 1:

Najar et al. have examined how observing others' actions affect one's own value-based decision-making in the context of instrumental learning. Fitting various computational models to the behavioral data and comparing their goodness of fit, they have demonstrated that observation of others' actions directly affects the observer's value function in a computationally simple way. They have further shown that the imitation process is modulated by how much the others' actions are informative (i.e., expertise or skill-level). I believe the authors have addressed an important issue in human social decision-making with a solid experimental design and careful data analyses. Their findings would therefore provide significant insights into psychological mechanisms underlying human social decision-making. On the other hand, I am not sure whether the present study could be of wide interest not only to researchers who are interested in human social decision-making.

We thank the Reviewer for the positive evaluation and the constructive comments.

R1.1: The authors have demonstrated that one class of the models (i.e., value-shaping) outperformed the other classes (i.e., decision-bias and model-based imitation). What is the qualitative difference among the three types of the models? In other words, what types of behavioral patterns in this experiment and the real world can be "exclusively" explained by the winning model? To answer this question by model-based and/or model-free analyses might increase the novelty and significance of the study.

This is indeed a very good point. Motivated by the Reviewer's request, we performed posterior predictive checks to identify specific behavioural signatures of the compared models. The analysis capitalizes on the specific feature of our experimental design that is meant to detect the accumulation of demonstrations and the propagation of imitation over several trials. As two private trials can be interleaved with a varying number of observational trials, we can measure the effect of accumulation of several consecutive demonstrations. In the same way, because observational trials can be interleaved with a varying number of private trials, we can measure the effect of the propagation of imitation over several consecutive private choices. Specifically, we measured <u>*accumulation*</u> as the difference in behavioural imitation rates between two successive demonstrations $d_{t-1}$and d_{t-2} preceding a private choice c_t. On the other hand, the <u>*propagation*</u> was measured as the difference in behavioural imitation rates between two successive private trials $c_{t+1}$and c_{t+2}following a demonstration d_t. Behavioural imitation rates were computed as the average number of trials where the choice $c_i$was equal to the demonstration d_j. The adjective 'behavioural' is added to define this metric to differentiate it from the model parameter also called imitation rate (for analogy with the learning rate).

We performed posterior predictive checks by comparing the predictions of each simulated model (using the best fitting parameters) to the real data (cf. figures **Fig F1** and **Fig F2**). In addition to the *value-shaping* (VS) and *model-based* (MB) models, we included two variants of *decision-biasing* (DB). The first variant, DB1, corresponds to the "state-of-the-art" model that

has been widely considered in the literature, which, in a sense, implements a 'pure' decision-biasing process (Burke et al. PNAS,2010). The second variant, DB6, is our improved version of DB1, which implements accumulation, and was the winning model within the *decision-biasing* family of models.

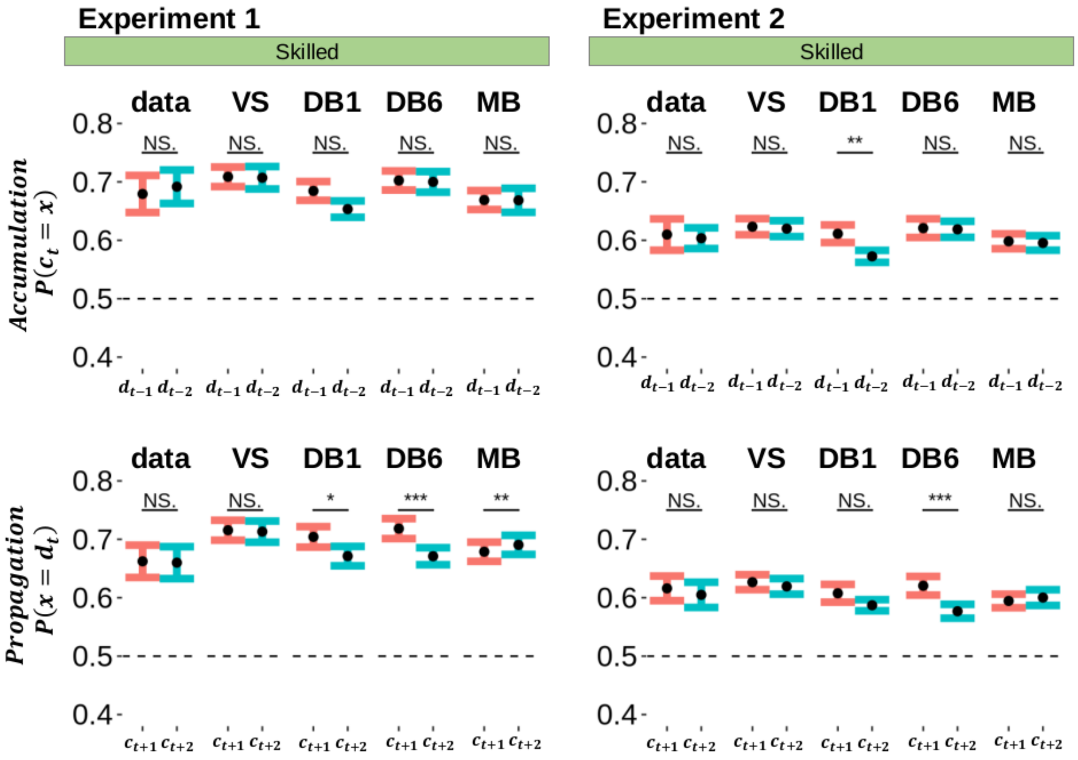

Fig F1. Model properties: Accumulation (top row): behavioural imitation rate calculated as a function of the two preceding demonstrations ($d_{t-1}$and d_{t-2}). Propagation (bottom row): behavioural imitation rate calculated as a function of two consecutive choices ($c_{t+1}$and c_{t+2}). Paired t-test *p<0.05, **p<0.01, ***p<0.001.

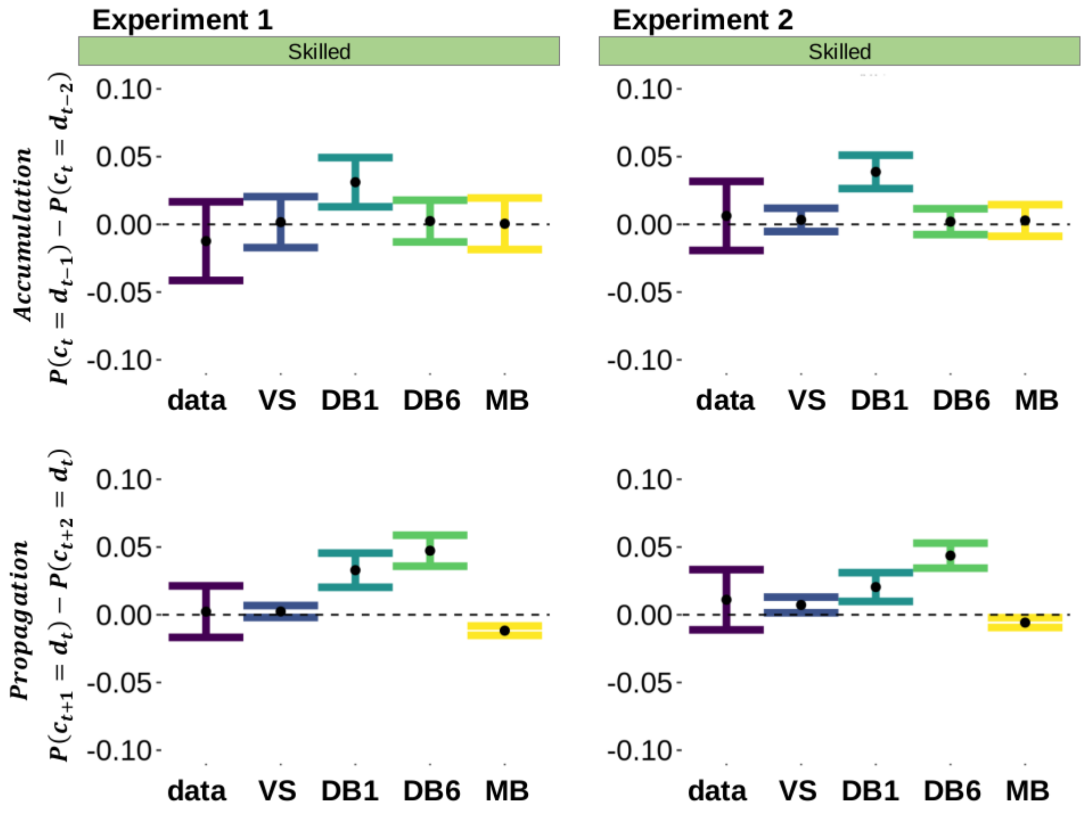

Fig F2. Difference in the behavioural imitation rate calculated between the two preceding demonstrations (accumulation: top row) and calculated between two consecutive choices (propagation: bottom row). The first metric rejects the DB1 model, while the second metric rejects both DB1 and DB6 models. The VS and MB models' average predictions are within the observed ranges, with the VS model being slightly closer to the data.

As expected, we found that DB1 failed to capture the effect of accumulation of demonstrations, as the demonstration at $d_{t-2}$was associated with a smaller behavioral imitation rate compared to the demonstration at d_{t-1}. This effect was corrected by allowing a short term accumulation of the demonstrations in DB6, which made it closer to real data, to the same extent as VS and MB. However, both DB1 and DB6 were not capable of reproducing the propagation of demonstrations over trials, as the demonstration at $c_{t+2}$was associated with a smaller behavioral imitation rate compared to the demonstration at c_{t+1}. Both VS and MB made good predictions about the propagation effect, but VS was closer to real data compared to MB.

In summary, we did find different behavioural signatures of the different models in relation to a specific feature of our design: allowing for several consecutive choices and demonstrations. All models (except standard *decision-biasing*) made good predictions about accumulation. However, *value-shaping* was the best model in predicting the effect of propagation. Thus, posterior predictive checks "falsify" the *decision-biasing* models, and consolidate the model comparison results about *value-shaping* being the "winning" model.

We included this new analysis in the manuscript. We added a new section “Model properties” **page 7**, a new figure **Fig 4**, and a Supplementary **Fig S6.** We list here the main changes made to the text.

Results (**pages 6-7**):

> We analyzed model simulations to identify specific behavioural signatures of our imitation models. This analysis capitalizes on the specific feature of our experimental design, namely the fact that we allowed for several observational or the private trials to be presented in a row (while keeping their number the same). This feature allowed us to assess the accumulation and the propagation of social information over several consecutive trials. We restricted the analysis to the skilled demonstrator condition, as we already showed that imitation is suppressed in the unskilled demonstrator conditions. We defined the behavioural imitation rates as the average number of trials where the choice c_i was equal to the demonstration d_j. Behavioural imitation rates were higher than chance in all cases (Supplementary Fig S6). We defined accumulation as the difference in behavioural imitation rates between two successive demonstrations $d_{t-1}$and d_{t-2} preceding a private choice c_t. A *Learner* that takes into account only the last demonstration should display a positive differential. Similarly, propagation was measured as the difference in behavioural imitation rates between two successive private trials $c_{t+1}$and c_{t+2}following a demonstration d_t. A *Learner* that uses demonstrations only to bias exploration should display a positive differential.
>
> We compared the simulation of each model to the empirical data (Fig 4). Models were simulated using the set of the best fitting parameters. In addition to the value-shaping (VS) and model-based (MB) models, we included two variants of decision-biasing (DB) (cf. Methods). The first variant, DB1, corresponds to the standard implementation of decision biasing that has been widely considered in the literature, which implements a ‘pure’ decision-biasing process [6,15]. The second variant, DB6, is our improved version of DB1, which includes the accumulation of demonstrations over consecutive observational trials in a temporary memory trace, and was the winning implementation within the decision-biasing family of models (cf. supplementary Fig S2).
>
> As expected, we found that the standard model (DB1) failed to capture the effect of accumulation of demonstrations, since the demonstration at d_{t-2} was associated with a smaller behavioral imitation rate compared to the demonstration atd_{t-1}. This effect was corrected by allowing a short term accumulation of the demonstration in DB6, which brought it closer to real data, to the same extent as VS and MB. However, both DB1 and DB6 were incapable of reproducing the propagation of the demonstration over trials: the private choice at c_{t+2} was associated with a smaller behavioral imitation rate compared to the private choice at c_{t+1}. Both VS and MB made good predictions about the propagation effect, but VS was closer to real data compared to MB.
>
> In summary, we did find different behavioural signatures of the different models in relation to a specific feature of our design: allowing for more than one consecutive private or observational trials. All models (except the standard decision-biasing: DB1) were capable to reproduce the effect of accumulation. However, neither version of *decision biasing* was capable to reproduce the effect of propagation. Thus, model simulations “falsify” the decision-biasing models, and consolidate the model comparison results to support value-shaping as the “winning” model.

Finally, in addition to analysing these distinctive features of our model given our design, we also checked whether our winning model was capable of capturing the observed behaviour in an unbiased manner, by computing the correlation between observed and simulated data-points. We found that the VS model captured well both between-trial and between-subject variance, as all the correlations displayed slopes very close to one, intercepts very close to zero (see Supplementary Fig S7 and Fig S8).

R1.2: The data indicate that participants infer the expertise of a Demonstrator. It would be interesting to construct computational models that incorporate learning about others' expertise. As far as I know, it remains elusive whether and how learning from others' action (e.g., Burke et al., 2010 and the present study) is integrated with learning about others' expertise (e.g., Boorman et al., 2013). Examination of this issue could reinforce the novelty and significance of the study. In relation to this issue, I don't agree with the authors' claim that participants do not build a model of the Demonstrator. They clearly have a model of the Demonstrator, and it modulates the participants' imitation behavior.

We thank the Reviewer for their relevant suggestion, clear and constructive expression of their disagreement and for pointing out Boorman et al. 2013. Indeed, the *payoff-based heuristic,* where the level of imitation depends on the performance of the demonstrator, is amongst the various social learning strategies that have been documented in the literature [Schlag, *Journal of economic theory,* 1998; Kendal et al., *Journal of theoretical biology*, 2009; Boorman et al., *Neuron,* 2013]. However, in our work the performance of the demonstrator was not directly accessible to our subjects as only the demonstrator's choice but not their outcomes were shown. The main reason for this is that we were interested in studying the effect of imitation and not vicarious reinforcement which has been reported to override imitation [Selbing et al., *Cognition,* 2014; Safra et al., *PLoS computational biology,* 2019].

However, even though the demonstrator's reward function is not directly accessible to the subject, we postulated that the subject could use their own reward function as a proxy for outcome to assess the demonstrator's skill on the task. So, the value of an observed action would be given by the subject's own Q-values. In this way, the subject is able to track the *inferred skill* of the demonstrator by relying only on trial-to-trial *agreement* between them , without building any explicit model of the demonstrator. Indeed, several previous studies have provided empirical evidence for subjective value of agreement in value-based decisions on matters of taste (Campbell-Meiklejohn et al. *Current Biology*, 2010; Izuma & Adolphs, *Neuron*, 2013). The model we propose here builds on the previous studies to provide a subjective, and importantly, still model-free way to infer the demonstrator's skill.

We tested several implementations of this hypothesis by using either raw Q-values, softmax outputs or the greedy decision of the subject's Q-values (argmax). The winning implementation was the one where the imitation rate is dynamically modulated depending on whether or not the observed action maximizes the learner's current Q-values. The imitation rate is first initialized to zero for every state (i.e., pair of cues), then updated trial-by-trial using an auxiliary learning rate (free parameter) that measures how fast a subject learns about the skill of the demonstrator.

We fitted this model (which we call meta-VS) to our behavioral data and compared it to the value-shaping model (VS). Model comparison shows that value-shaping with meta-learning (meta-VS) explains the subject's choices better than the standard value-shaping model VS (see **Fig F3a**). In order to compare the average imitation rates over the skilled and the unskilled conditions, we performed posterior predictive checks by simulating the meta-learning model (meta-VS) using the fitted parameters. We were able to reproduce the significant modulation of the imitation rates, which mirrors the results we found by fitting two separate learning rates for the skilled and unskilled demonstrators in the VS model (see **Fig F3b**).

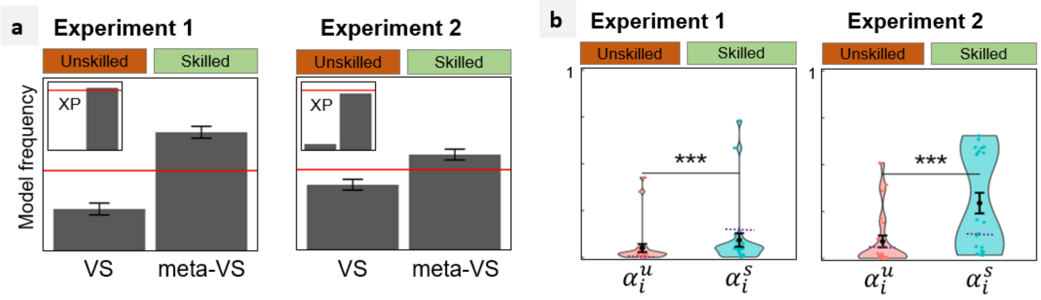

Fig F3. Meta-learning model. (**a**) Model comparison between the standard value-shaping model (VS) and value-shaping with meta-learning (meta-VS). ‘Xp’ denotes exceedance probability (**b**) The analysis of the average imitation rate over the experiment replicates the modulation effect with respect to the performance of the demonstrator (skilled vs. unskilled). The black dots represent the average imitation rate found by fitting two different parameters in the VS model.

We also analyzed the evolution of the imitation rate over the time course of the experiment. The meta-VS model was successful in generating different modulations of the imitation rate for the skilled and unskilled demonstrators (see **Fig F4**).

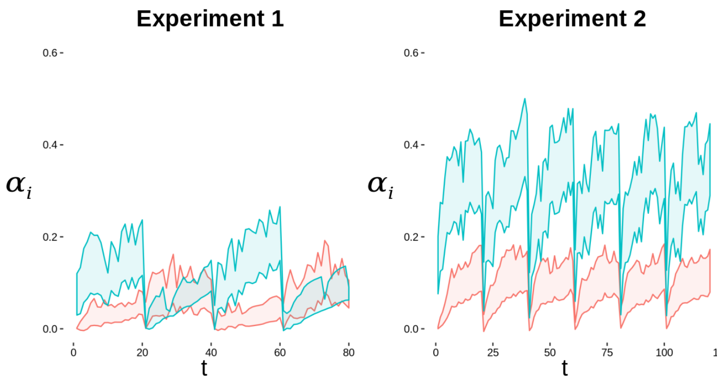

Fig F4. Average time course over the experiment of the imitation rate in the meta-learning model. Blue lines represent the values for the skilled Demonstrators, red lines represent the values for the unskilled Demonstrators. The upper line represents the mean+s.e.m., the lower line represents the mean-s.e.m.

The inclusion of the meta-learning model led to many changes in the manuscript (including the title, the abstract and current **Figure 6**) and significantly improved the content of our work. We are therefore grateful to the reviewer for their helpful disagreement. Here, we list the main changes:

Results (**pages 8-9**):

In order to account for the modulation of imitation as a function of the Demonstrator's skill, we implemented a meta-learning model where Learners assess the performance of the Demonstrator and adapt their imitation accordingly.

Since the Demonstrator’s choice outcomes and thereby, reward function is not directly accessible to the Learner, we postulated that the subject uses their own reward function as a proxy for outcome to assess the Demonstrator’s skill on the task. The imitation rate is first initialized to zero for every state, then updated trial-by-trial using an auxiliary learning rate (see “Methods”). In this framework, if the Demonstrator chooses the action (or option) that the Learner currently believes is the best, in other words, if the demonstrator agrees with the Learner, the imitation rate should increase. The converse is true when the Demonstrator chooses the action that the Learner currently believes is not the best By comparing the agreement between Demonstrator’s actions to their own value function, the Learner is able to track a subjective inferred skill of the Demonstrator by relying only on his own evaluation of the task, without building an explicit model of the Demonstrator. This would provide an effective, and yet model-free, way to infer the Demonstrator’s performance.

We fitted this meta-learning model (which we call meta-VS) and compared it to the VS model. Results show that value-shaping with meta-learning explains the subject's choices better than the standard value-shaping model in Experiment 1 (MF: 0.74, XP: 0.99), and Experiment 2 (MF: 0.59, XP: 0.9) (Fig 6.a). We analysed the trial-by-trial estimates of the imitation rate averaged separately for SD and the UD blocks and found that we were also able to reproduce the observed significant modulation of the imitation rates (Exp 1: Wilcoxon signed-rank test: V = 0, p=1.192e-07; Exp 2: Wilcoxon rank-sum test: W=88, p=0.0001742) (Fig 6.b).

Discussion (**pages 11-13**):

However, in our task, imitation rates could not have been modulated by such hypothetical comparison of the outcomes because we did not present the Demonstrator's outcome. We proposed (and tested) an alternative modulation process that is based on the evaluation of the Demonstrator's choices in the light of the Learner current preferences. The basic intuition is that if the demonstrators’ choices agree with what the learner would have done, the learner starts to ‘trust’ the Demonstrator. Our model is inspired by several previous empirical findings showing that the human brain’s reward network is responsive to agreement in decisions on matters of taste that have (by definition) no correct outcome [31,32]. Thus, the learner treats the Demonstrator's choice as a surrogate reward (value-shaping) preferentially when they present an overall high agreement rate.

Consistent with this hypothesis, Boorman et al. [33] showed that in a market prediction task learning of the expertise of another agent (the role akin to the Demonstrator in our task) was accelerated when she/he expressed a similar judgement. The meta-learning model managed to capture the adaptation of the imitation rate to the type of the Demonstrator across the three experiments. A caveat of our implementation is that it supposes that the initial imitation rate is zero, a simplifying assumption that may not reflect many real life situations, where imitation is the

primary source of information. It should be noted that our meta-learning framework could easily be extended by assuming an additional free parameter determining the baseline imitation rate (we tested this implementation in our data, but it was rejected due to the additional free parameter).

In Experiment 3 we were able to find a significant modulation of imitation only when the participants were informed about the IQ of the Demonstrator. This finding indicates two things. First, for the case when the Demonstrator's IQ was hidden, the performance gap between UD (~0.65 correct choice rate) and SD (~0.85) was not large enough to endogenously modulate imitation. Second, when the Demonstrator's IQ was visible, imitation could also be controlled by exogenous information about cognitive abilities. This finding is consistent with many studies showing that reputational priors shape learning and decision-making at the behavioural and neural levels [34,35].

Methods (**page 17**):

Meta-learning:

To account for the dynamic modulation of the imitation rate we designed a meta-learning model. The core idea of the model is that the skill of the Demonstrator is inferred by comparing her choices to the current knowledge of the Learner.

The imitation rate α_i in the meta-learning model (meta-VS) is first initialized at zero for every state (i.e. pair of cues). Then, $\alpha_i$is dynamically modulated on a trial-by-trial basis depending on whether or not the current observed action maximizes the learner's Q-values. The modulation is performed using an auxiliary learning rateα_m that measures how fast a subject learns about the approximate competence of the Demonstrator:

$$\alpha_i(s) \leftarrow \alpha_i(s) + \alpha_m * (\tau - \alpha_i(s)),$$

where s represents the current state (pair of cues), and

$$\tau = \begin{cases} 1 & \text{if } Q(d) = max(Q(d), Q(\bar{d})) \\ 0 & \text{otherwise} \end{cases}$$

Note that, in meta-VS, α_m is a free parameter whereas α_i is not. The imitation rate is then used for updating the Learner's value function through value-shaping using equations 7 and 8.

R1.3: It is difficult for me to understand details of the computational models. The authors should provide full descriptions of the models. Short descriptions combined with schematic diagrams are not sufficient to reproduce the models in future studies.

We thank the Reviewer for pushing us to further improve model description. In the revised version of the manuscript we included, in addition to the bow and arrow description figure and the verbal description, the key equation of the VS model in the "*Model comparison*" results section (**page 5**):

Finally, in the value shaping model (VS), Demonstrator's choices directly affect the value function of the Learner using the following equation:

$$Q(d) \leftarrow Q(d) + \alpha_i \times [1 - Q(d)],$$

where d is the action chosen by the Demonstrator, Q the value function of the Learner, and α_i an imitation rate. In other terms, the VS model assumes that the Demonstrator's choice is perceived as a positive outcome (or surrogate reward).

As in the previous version of the manuscript the full equations are provided in the methods and in the **Figures 7** and **8**, where we describe the different implementations of the models. Finally, in order to be completely transparent and to facilitate reproducibility, we uploaded the data and the source code on the Human Reinforcement Learning team GitHub:
https://github.com/hrl-team/mfree_imitation

R1.4: Having said that, I would like to suggest other possible models worth tested.

We appreciate the Reviewer's suggestions. First, we would like to note that the main model comparison includes four models only, for clarity and conciseness. However, this only represents the "tip of the iceberg", because these final models were selected from no less than 21 different competing models (presented in the "Computational modeling" section, **page 16**) aimed at identifying the best possible implementation of each family of models. We present additional results and arguments in the points below.

R1.4a: [1] Baseline model. In the current model, lambda reflects only the last action. Another way is to assume accumulated choice-trace affects decision-making (e.g., Akaishi et al., Neuron, 2014). What happen if the latter formulation is employed? Note that the latter formulation of a choice auto-correlation include the former original formulation as a special case.

We thank the Reviewer for pointing out Akaishi et al 2014, and for suggesting the investigation of more general implementations of choice auto-correlation. Indeed, we had implemented choice-autocorrelation in our models, even though this question is orthogonal to our main question, which is about imitation. We demonstrated this claim in the initial version of the paper, by showing that our model comparison results were robust against the implementation details of the model space, i.e. with and without choice auto-correlation, with and without symmetric value update for private learning, with and without allowing for negative imitation learning rates (cf. Supplementary **Fig S3;** reported also below as figure **F5**). This was done by implementing 5 additional variants of the final model space, bringing the total amount of compared models to 26.

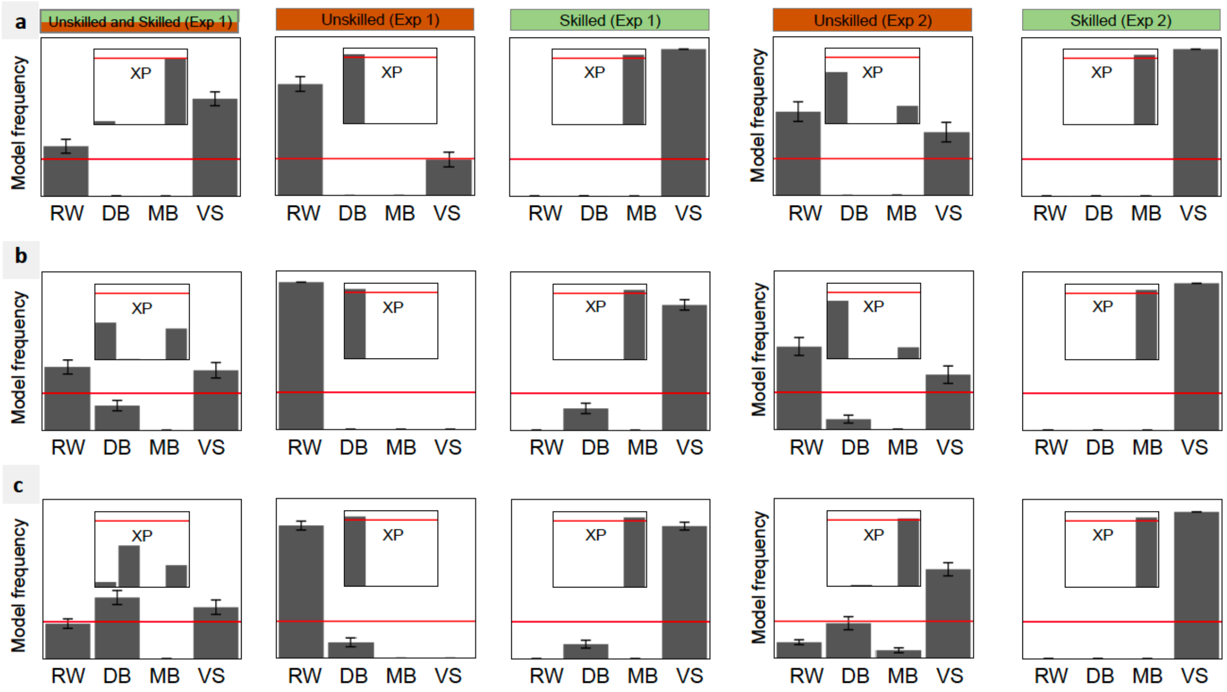

Fig F5. Model comparison results are robust amongst different implementations of the model space. a) Model space implementation without choice auto-correlation parameter. b) Model space implementation without symmetric value update for private learning. c) Model space implementation allowing for negative imitation learning rates. Note that in Exp 2, when allowing for negative learning rates, the winning model in the UD condition is no longer RW, but VS.

R1.4b: [2] Decision biasing models. How about a model, in which participants keep track of the Demonstrator's accumulated choice-trace (rather than the current action d) and the choice-trace works on the action-selection process?

The Reviewer suggests a model where the choice-trace of the Demonstrator accumulates across trials and biases action selection. In our task, there are two possible ways to conceive choice-trace accumulation: either across each block of consecutive demonstrations or across all demonstrations within a learning context (pair of symbols).

Concerning the first possibility, accumulation across consecutive demonstrations is precisely what we have done in DB6: the winning implementation of the *decision-biasing* model. Indeed, it is true that in the original formulation of the *decision-biasing* model, DB1, the action selection bias was only based on the last demonstration [Burke et al. 2010]. However, to make a stronger case in favour of *value-shaping*, we first compared several implementations of *decision-biasing*, including three implementations, DB4, DB5 and DB6, where the choice trace accumulated across consecutive demonstrations (cf. "Computational modeling" section, **page 17** and **Fig 7** in the paper, reported below as **Fig F6**). The winning implementation within the decision-biasing family was DB6, which implemented accumulation across each block of consecutive demonstrations.

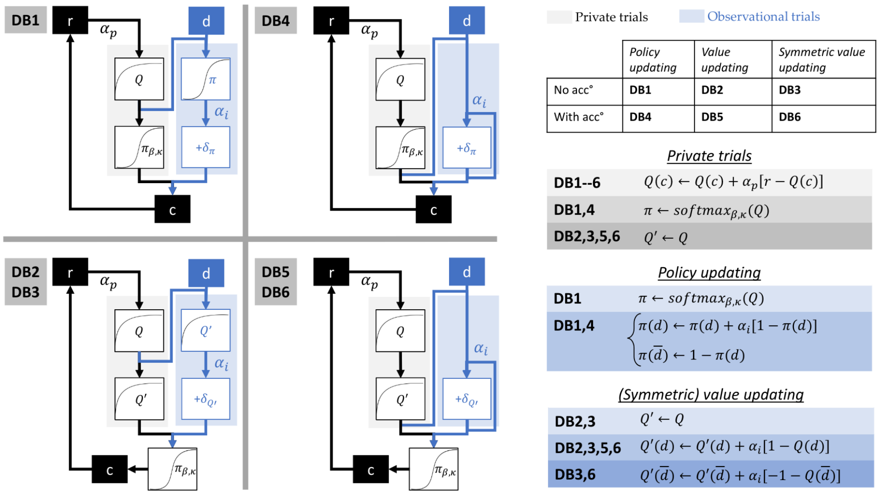

Fig F6. Six implementations of decision biasing. In DB1, demonstrations bias Learner's actions via policy update. DB2 and DB3 implement the same mechanism through value update and symmetric value update. DB4, DB5 and DB6 are equivalent to respectively DB1, DB2 and DB3 while allowing for the accumulation of successive demonstrations. This is done by removing the first step of the update where π or Q' is derived from Q. Accumulation is depicted in the diagrams by the loop within observational trials (see also **Supplementary Fig S2** for the results pertaining this preliminary model comparison).

Regarding the second possibility, accumulation across all demonstrations within a learning context is precisely implemented by the several variants of *model-based* imitation, where the model of the demonstrator is built either as an accumulated choice-trace (represented by the Q-values in MB4, MB5, MB6, MB7, MB8 and MB9) or a normalized choice-trace (represented by the policy in MB1, MB2 and MB3) (cf. "Computational modeling" section, **page 18** and **Fig 8** in the paper, reported below as **Fig F7**). The winning implementation within the model-based family is MB9, accumulates the demonstrator's choice-trace as Q-values via symmetric value update (cf. Supplementary figures **S2** and **S5**).

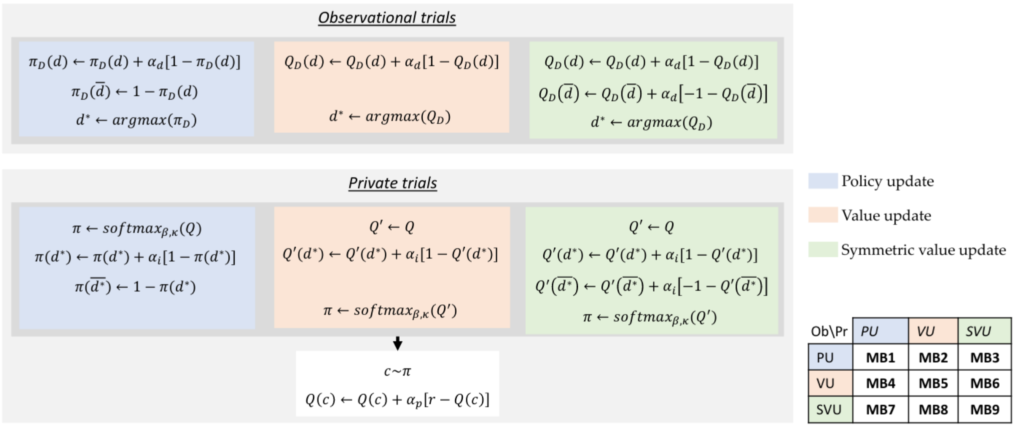

Fig F7. Nine implementations of model-based imitation. In observation trials, demonstrations are used for building a model of the Demonstrator (π_D or Q_D). This is done either through policy update (MB1, MB2 & MB3), value update (MB4, MB5 & MB 6), or symmetric value update (MB7, MB8 & MB9). In private trials, the model of the Demonstrator

is used for biasing Learner's actions through either policy update (MB1, MB4 & MB7), value update (MB2, MB5 & MB8), or symmetric value update (MB3, MB6 & MB9).

R1.4c: [3] Model-based imitation models. How about a model, in which model-based inference about the Demonstrator's value function affects the participant's value function (rather than decision-making)?

Even though we concede that a proper model comparison analysis should be maximally inclusive in order to disentangle the effects of different computational elements, we believe that model inclusion must be done in a principled way. We did not include this hybrid model because we found it conceptually redundant. The basic idea behind model-based imitation is that an imitator represents the preference of another agent as separate from her own. This assumption relies precisely on the fact that the two value functions are separate. As soon as one value function affects the other, the notion of a 'separate model for the demonstrator" does not hold anymore.

Moreover, when implementing *model-based* imitation, there are two different "moments" where the influence of the demonstrator's model could take place: either at demonstration time, or at decision time. In our implementations, the influence happens at decision time when the subject has to make a choice. If we do the same for the proposed model, the influence of the demonstrator's model would infinitely increase each time the subject makes a choice, without any intervention from the demonstrator. This effect does not make sense as demonstrations should only accumulate over demonstrations, not during private trials. This is also falsified by our observations as we do not observe any increase of imitation over consecutive choices (cf. **Fig F1**).

The second place where the demonstrator's model could influence the *Learner* is at demonstration time, and this is exactly the place where the model of the demonstrator is updated. So, we would update the demonstrator's model and use it right away to update the learner's value function. This would be redundant as it would have the same effect as *value-shaping* in the long run. Anyway, as the reviewer raised the point, we tested this implementation, referred to as *model-based value-shaping* (MBVS), and it performed worse than both *value-shaping* (VS) and *model-based imitation* (MB), cf. **Fig F8**.

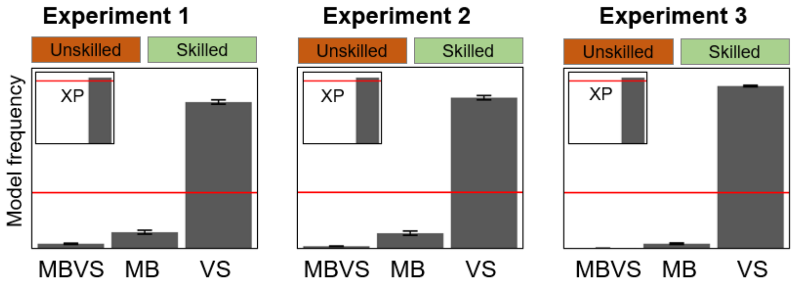

Fig F8. Model comparison between value-shaping (VS), model-based imitation (MB) and model-based value-shaping (MBVS). We replicate this result also in the Experiment 3, that will be introduced later in the Rebuttal letter.

R1.5: I am not fully convinced by the authors' claim about egocentric bias. They have compared learning rate for participants' own REWARD and that for the Demonstrator's ACTION. The differential learning rates observed in this study can be attributed into the difference between learning from REWARD and from ACTION (rather than difference between self and other).

The Reviewer raised a fair point. We conceived the concerned paragraph of the discussion not as establishing a claim, but rather as a possible interpretation of the findings, to be addressed by further research. We amended the introduction and the results section and we downtoned the discussion to make our view clearer and take into account this alternative interpretation.

Introduction (**pages 3-4**):

> We also analyzed the parameters of the best winning model to determine whether or not, in the context of reinforcement learning, imitation more weight is given to information derived from oneself compared to the other [13].The comparison of the private reward learning rate with the imitation learning rate was overall consistent with more weight given to privately generated information.

Results (**page 7**):

> We first assessed whether subjects put more weight on their freely obtained outcomes or on the choices of the Demonstrator. We found the private reward learning rates were significantly larger compared to the imitation learning rates (Fig 3.b). This was true in Exp 1 (Wilcoxon signed-rank test: V=300, p=1.192e-07). In Exp 2, the difference between private and imitation learning rates was even more pronounced when confronted with an Unskilled Demonstrator (Wilcoxon signed-rank test: V=251, p=1.431e-06), and still detectable when facing a Skilled Demonstrator (Wilcoxon signed-rank test: V=200, p=0.01558).

Discussion (**page 12**):

> This difference could derive from the fact that the Demonstrator's action, as a proxy of reward, implies an additional degree of uncertainty (one will never know whether the Demonstrator obtained a reward, after all). This difference could also derive from an egocentric bias, where self-generated information is systematically over-weighted.

R1.6: I would like to make sure the model-fitting procedures in this study. As far as I understand, for the model comparison the authors fit each model to the behavioral data by minimizing negative log likelihood (i.e., maximum likelihood estimation), while the parameter estimation is performed based on log model evidence (derived by Laplace approximation). Why did they use different ways for model comparison and parameter estimation? This looks weird. Furthermore, P(data | model) is "model evidence", not "maximum likelihood". Moreover I believe AIC cannot be fed into Bayesian model comparison. The authors should double-check.

Parameter estimation was made twice at the subject level. First by maximizing log likelihood. Second, by maximizing the posterior probability of the data given the model and the parameters. Model selection was made at the group level by maximizing the posterior probability of the models using subject-specific AIC scores, which were itself derived from the maximum likelihood. On the other side, to analyze and compare the model parameters, we relied on parameters estimated maximizing the posterior probability.

Comparing subject-specific parameters estimated by maximizing the posterior probability is a standard approach in the literature [Daw et al. *Neuron*, 2011; Gershman et al., *Psy Rev Bul*, 2015; Palminteri et al. *Plos CB* et al., 2016; 2017]. The advantage of these parameters, in comparison to parameters obtained maximizing log likelihood, is that it generally avoids degenerate parameter estimation by relying on weakly informative priors. Nevertheless, in our case, we obtain the same conclusions when comparing the learning and imitation rates using maximum likelihood instead of maximum a posteriori (cf. **Fig F9**).

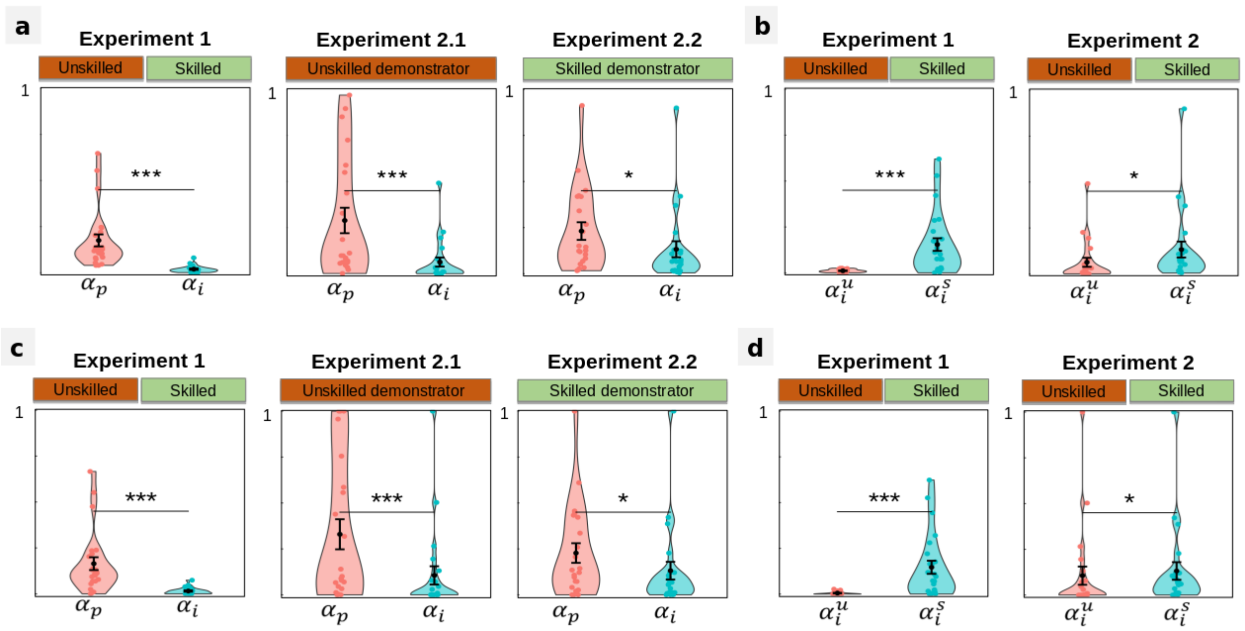

Fig F9. Parameter analysis using maximum likelihood and maximum a posteriori estimations. (**a**) and (**b**) maximum a posteriori estimation. (**c**) and (**d**) maximum likelihood estimation. Even though the distributions are slightly better shaped for the maximum a posteriori estimation, the same statistical conclusions hold for both estimation methods.

Regarding model selection, we follow a Bayesian approach which relies on subject-specific log model-evidences to compute the exceedance probability of each model (Stephan et al., *Neuroimage,* 2009). In our work, we use the AIC (derived from the log-likelihood) as an approximation of the model log-evidence for each subject, which is a common procedure in group-level Bayesian model selection (Stephan et al., *Neuroimage,* 2009). The AIC can indeed be used with the VBA toolbox in the same way as any other model log-evidence approximation:
https://muut.com/i/vba-toolbox/questions:vba-groupbmc-script
https://mbb-team.github.io/VBA-toolbox/wiki/VBA-output-structure.

Various approximations of the model log-evidence may have different performance depending on the experimental setting and computational models under consideration. To decide which approximation to use, we follow a principled way based on model recovery (i.e. the capacity to retrieve the correct model in simulated datasets where the ground truth is known). AIC provided good model recovery results, while log-likelihood, and log posterior probability show poor model recovery performance (cf. **Fig F10**). We added this information in the supplementary materials (Supplementary **Fig S8**).

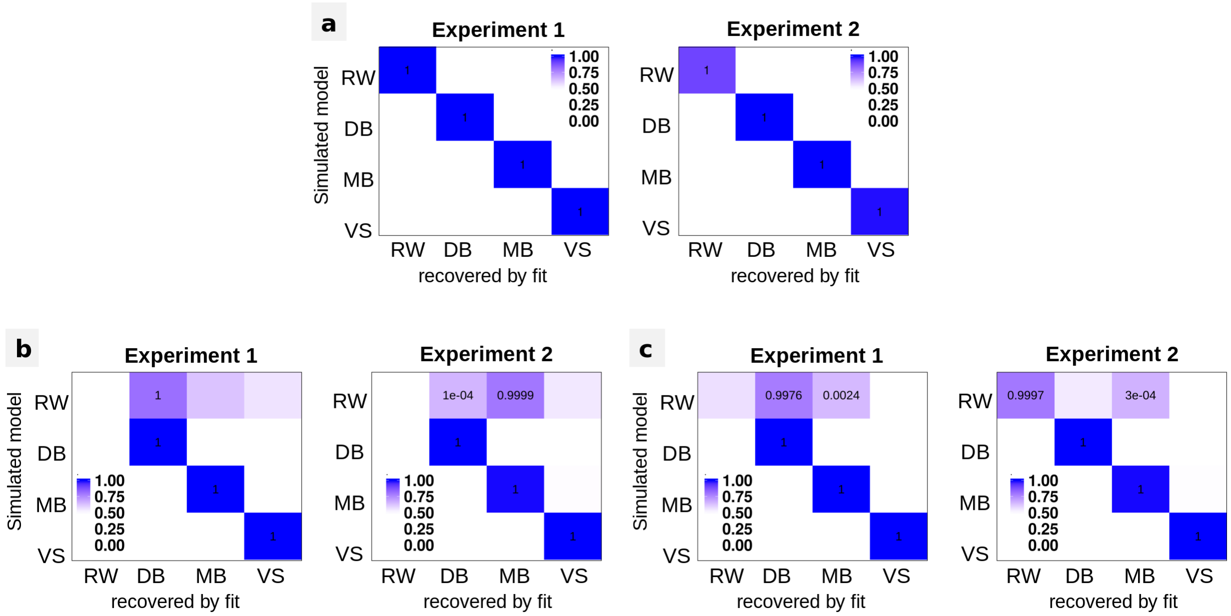

Fig F10. Model recovery using different approximations of model log-evidence for the main computational models (DB=DB6, MB=MB9) and simulating the experimental setting of Experiment 1 and Experiment 2. We tested different approximations of the model evidence that we fed to the VBA toolbox. (**a**) AIC. (**b**) log-likelihood. (**c**) log posterior probability. Only AIC displayed a good parameter recovery (meaning that the winning model is the model actually used to generate the synthetic data).

We made these points clearer in the revised version of the manuscript (Methods section, **pages 18-19**):

> We selected the AIC as a model comparison metric after comparing its performance in model recovery along with other metrics such as the LPP (see below).
>
> …
>
> While the LPP, in principle, could be used to compare models (as it integrates the probability of the parameters, therefore penalizing a higher number of parameters), it is usually not a very stringent criterion (especially when the parameter priors are weakly informative). Accordingly, in our case, it did not give a very good parameter recovery (see Supplementary Fig S8).
>
> …
>
> Our model recovery analysis confirmed that, in our task and for our models, the AIC is a good criterion, while LPP is not.

Reviewer #2:

R2.0: Imitation as a model-free process in human reinforcement learning
In this manuscript, the authors aim to elucidate the computational underpinnings of imitation, defined as using another's actions a source of information in a probabilistic two-armed bandit task, in human reinforcement learning. The authors tackle an important question in a methodologically rigorous manner. As such, I find the manuscript interesting, timely, and well written. However, given the scope of the question ("the exact computational implementation of how social signals/imitation influence human reinforcement learning", paraphrased from the abstract), and the quality level of the journal, it is my opinion that both additional data and analysis are required for providing general and conclusive answers to the research question. In short, I think the authors should be more ambitious in answering open questions they themselves identify. I outline my concerns and suggestions below, beginning with what I consider more major points.

We thank the Reviewer for the positive evaluation, the constructive comments and the encouragement. We note we removed the word 'exact' from the abstract.

R2.1: Reward shaping/policy shaping/value shaping: The authors appropriately acknowledge (page 7) that the single-step experiment does not allow distinguishing whether imitation shapes value, reward, or policy, which instead would require a multi-step design. Given that the goal of the study was to determine the algorithmic implementation of imitation, this remains an important open question. I don't understand why the authors did not attempt to address this question by also running a suitable multi-step experiment. Aside from being novel on its own right (I am not familiar with any multi-step designs in human social RL), such an experiment would allow addressing the important open question, and (possibly) confirm that the results generalize outside of the confines of the classic Burke et al. (PNAS, 2010) design.

We agree on the importance of generalizing our results to other experimental settings; and we share the Reviewer's interest towards the reward/value/policy shaping question that we raised in the discussion of our paper.

Regarding the generalization to other experimental settings, we first would like to mention that we did not just implement Burke's (2010) design, but we extended it into a new version, to measure both the accumulation and the propagation of imitation over several trials (see also **R1.1** and the new '*Model properties*' section in the results). However, even if in the original submission we reported results from two original experiments, we agree that this does not impede us from seeking additional validation for our claims. To further investigate the generality of our results, we included the analysis of an additional dataset from a recent paper (Vostroknutov et al.,*Scientific reports,* 2018). This is, to our knowledge, the largest dataset concerning imitation in human reinforcement learning we could access. Crucially,

Vostroknutov's experiment differs from ours in several aspects. For example, the outcome contingencies were not static, but followed a random walk. Also, demonstrator choices were actual choices of real participants (no deception was involved). The range of the Skills of the demonstrators was also very different as was the ratio between the number of private and observational trials. Finally, this new dataset includes N=302 subjects, which is almost 5 times more than our total sample size. The specifics of the new experiment are now presented in the Methods (**page 16**). We summarize the differences with our design in the following table (which also has been included in the manuscript as **Table 2** in the methods **page 16**):

Table: main differences between experiments. The variability on the Demonstrator's performance of Experiment 3 is given in standard errors of the mean.

	**Najar et al. (Exp 1)**	**Najar et al. (Exp 2)**	**Vostroknutov et al. (Exp 2)**
**N subjects**	24	44	302
**N trials**	400	300	300
**Ration observational / private trials (in social conditions)**	1:1	1:1	1:2
**Contingencies**	Stable (70/30)	Stable (60/40)	Random walk
**Demonstrators**	Computer	Computer	Real subjects
**Demonstrator skills (correct response rate)**	0.80 / 0.20 (within)	0.80 / 0.20 (between)	0.84±0.03<br>0.64±0.05<br>(between)

We are glad to report that the analysis of this third dataset replicates our main results. Model comparison favours the value-shaping mode (**Fig F11b** below). The average imitation rate was also found to be smaller compared to the average private learning rate.

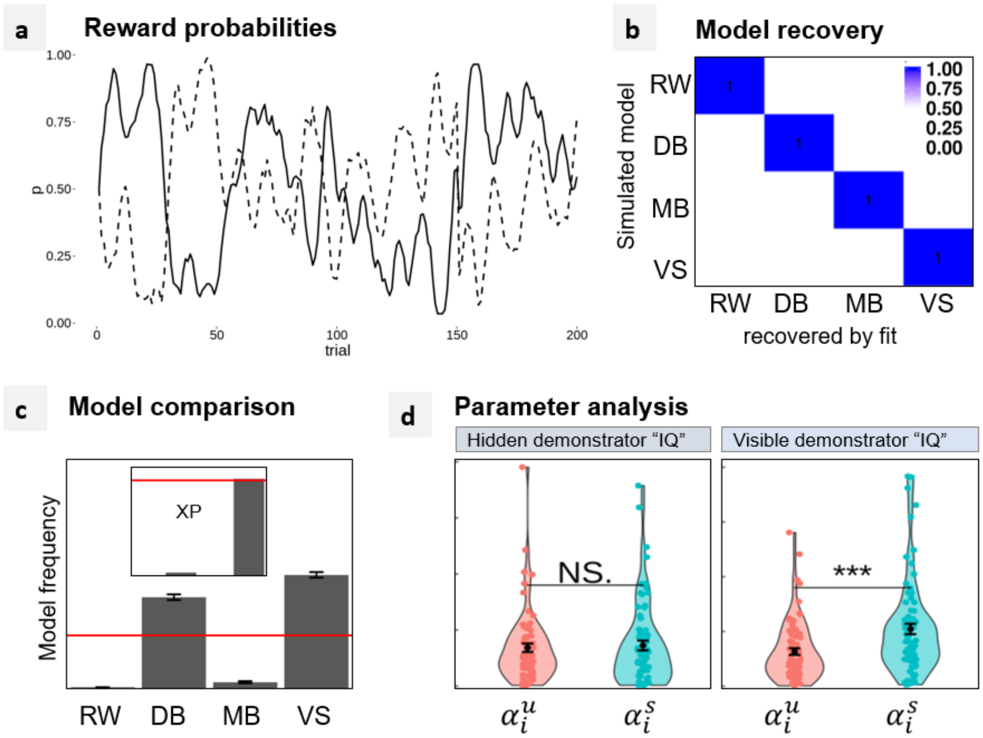

Fig F11. Replication of the main results on a third dataset (N=302). (a) Reward contingencies over trials for the two options (bold and dotted line, respectively) changed following a random walk. (b) Model recovery of the four main computational models using the AIC on simulated data using Experiment 3 specifications. The numbers written in the matrix indicate the exceedance probability, the intensity of the color indicates the posterior probability. (c) Model comparison. The value-shaping model VS is the winning model. (d) Parameter analysis: comparison of the imitation rate between subjects exposed to skilled and unskilled demonstrators. Leftmost panel: group for which the IQ of the demonstrator was not disclosed; rightmost panel: group for which the IQ of the demonstrator was disclosed.

Interestingly, the observational trials in Vostroknutov's experiment were drawn from choices of two previous subjects displaying different non-verbal IQ ('high' and 'low') and different average correct response rate (but note that not the same trials were shown to the participants, hence the variability in the demonstrator skills). Half of the subjects (N=152) were informed about the IQ of the demonstrator. Since the skilled / unskilled contrast was much smaller in Vostroknutov's experiment (64 vs. 84) this allowed us to test whether this smaller gap was sufficient to let us detect a modulation the imitation rate and whether the presence of exogenous cues (the information concerning the IQ) induced a modulation of imitation. The results indicate that when the IQ of the demonstrator is unknown there is no modulation of the imitation rate, suggesting that the gap in the Demonstrators' skill is not enough to induce a modulation. On the other side, when the IQ of the Demonstrator was known, we detected a significant modulation of the imitation rate, thus suggesting that exogenous cues may modulate imitation.

The inclusion of this third experiment led to many changes in the manuscript, which we list here. Results (**page 7**):

> To test the robustness of our results in another experimental setting and a larger cohort, we analyzed an additional dataset (N=302) from a recently published study [16]. This experiment, hereafter noted as Exp 3, differs from ours in several aspects (see Methods and Table 2). First of

all, the outcome contingencies were not static, but followed a random walk (Fig 5.a); and Demonstrator choices were actual choices recorded from two previous participants playing the exact same reward contingencies. One Demonstrator had relatively poor skill and the other relatively higher. The same choices were not necessarily displayed. As a result the observed Demonstrators's skills ranged from 0.53 to 0.92 in accuracy. First, we checked that using Exp 3 set up we got a good model recovery with respect to our main model space, allowing us to meaningfully compare the RW, MB, DB and VS models (Fig 5.b). Model comparison on the empirical data, replicated Exp 1 and Exp 2 results, showing that the VS model was the most plausible (MF: 0.53, XP: 0.97).

We then assessed whether subjects weighted their freely obtained outcomes more than the choices of the Demonstrator as they did in Exp 1 and Exp 2. Again, we found the private reward learning rates significantly higher compared to the imitation learning rates (Wilcoxon signed-rank test: V=42747, p< 2.2e-16). Finally, Exp 3 presented a novel feature such that half of the participants were informed about the IQ of their Demonstrator (N=150), while the other half vere not (N=152). In this experiment, the skilled Demonstrator (0.84±0.03 correct response rate) presented a higher non-verbal (IQ = 28/30 compared to the unskilled Demonstrator (0.64±0.05 correct response rate; IQ = 15/30). This configuration allowed us to test whether a smaller gap in the Demonstrator's skills (20% difference in the correct response rate) is sufficient to induce a detectable modulation in the imitation learning rate and whether the modulation is sensitive to exogenous cues signaling cognitive ability.

We submitted the imitation rate to a two-way ANOVA with IQ information (visible / hidden) and {Demonstrator's skill as a between-subject factors and we found a main effect (F(1,298)=8.501,p=0.00382) moderated by the IQ visibility factor (interaction: F(1,298)=5.248,p=0.02267). Post-hoc test confirmed that the imitation learning rates were significantly different only in the 'visible IQ' subjects (Wilcoxon rank-sum test: W=2092, p=0.0002356), but not in the 'hidden IQ' subjects (Wilcoxon rank-sum test: W=2634, p=0.7544) (Fig 5.d).

Discussion (**pages 9 & 11-12**)

Over three experiments, featuring different outcome contingencies and different ratios between private learning and observational learning trials, we found that imitation takes the computational form of value shaping, which implies that the choices of the Demonstrator affect the value function of the Learner.

...

In Experiment 3 we were able to find a significant modulation of imitation only when the participants were informed about the IQ of the Demonstrator. This finding indicates two things. First, for the case when the Demonstrator's IQ was hidden, the performance gap between UD (~0.65 correct choice rate) and SD (~0.85) was not large enough to endogenously modulate imitation. Second, when the Demonstrator's IQ was visible, imitation could also be controlled by exogenous information about cognitive abilities. This finding is consistent with many studies showing that reputational priors shape learning and decision-making at the behavioural and neural levels [34,35].

Methods (**page 14**):

The third dataset presented in this paper comes from a previously published study [16]. The main differences between our design and theirs are summarized in Table 2 and include: reward contingencies changed on a trial-by-trial basis (random walk); the Demonstrator choices consisted in recorded choices of previous participants playing exactly the same contingencies; they have an experiment with no social component (no deception was implemented); the ratio between observational and private trials was 1:2 (and not necessarily the same demonstrations were presented to the subjects); some participants (50%) received information about the I.Q. (raven matrices) of the Demonstrator. We refer the readers to the original publication for full details of the experimental task [16].

Regarding the reward/value/policy shaping question, we believe that the comparison between decision-biasing and value-shaping in a two-armed bandit task, is already interesting and complex enough to justify a paper *per se*. Infact, although similar observational learning tasks have been present in the literature for a while (in both their Pavlovian and instrumental versions: see Olsson et al. (2007), Burke et al. (2010)), this fundamental computational question has never been tackled explicitly and properly. Even recently, a review of the literature published in Nature Review Neurosciences [Olsson et. al, 2020], presented decision-biasing (in its original DB1 form) as the standard model of observational learning in the instrumental learning setting. This makes our paper even more timely and needed.

From another perspective, the way multi-step problems are computationally solved by humans is still an open question, as several possible models have been proposed in the literature, such as model-free learning [Tanaka et al; *Nature Neuro* 2004], model-based planning [Huys et al. *PNAS* 2015], or a combination of both [Daw et al. *Neuron*, 2012: Momennejad et al. *NHB*, 2017]. So, unlike two-armed bandit settings, there is still no consensus about what the baseline model over which we could build hypotheses about social learning mechanisms in multi-step problems should be.

For all these reasons, we believe that it is still too early for the community to be able to answer this question, and that an intermediate step, including our paper, is required before tackling this more general but more challenging question. Nevertheless, not addressing this question does not reduce the relevance of our findings about imitation being a model-free process, regardless of whether this happens at the level of the reward function, the value function or the policy.

R2.2: Adaptive modulation of imitation: The authors find, by means of separately fitting the skilled (SD) vs unskilled demonstrator (UD), conditions that people are able to adjust their level of imitation (i.e., people don't imitate in the UD conditions, as shown by the fit of the RW model). Furthermore, the authors discuss (pages 8-9) this in terms of a meta-learning process, and offer some suggestions for what information participants might use. Despite these observations, they do not, to my surprise, test these suggestions with additional modeling. I am therefore left rather unsatisfied with the modeling analysis, which is somewhat coarse (in fitting different within-participant conditions with different models). To me, a convincing account of the mechanisms underlying human imitation in RL would specify mechanisms that determine when people imitate (and thereby fit both

SD and UD with the same model in Exp. 1). I also assume that this would be the goal of the experimental design (why else would both SD and UD conditions be included?).

The reviewer here makes a very relevant suggestion (which was also made by Reviewer 1; see also **R1.2** for further details and the text added to the manuscript). Following the reviewer's request, we implemented a meta-learning model where subjects infer the skill of the demonstrator based on the agreement between the Demonstrators' action and their own subjective evaluation.

Since the demonstrator's reward function is not directly accessible (Demonstrator's outcomes were not shown) to the subject, we postulated that the subject uses their own reward function as a proxy to assess the demonstrator's skill on the task. So, the value of an observed action would be given by its agreement with the subject's Q-values. This would provide a subjective, but still a model-free way to infer the demonstrator's skill, without building an explicit model of the demonstrator.

We tested several implementations of this model, by using either raw Q-values, softmax outputs or the greedy decision of the subject's Q-values (argmax). The winning implementation is the one where the imitation rate is dynamically modulated, depending on whether or not the observed action maximizes the learner's current Q-values. The imitation rate is initialized to zero for every state (i.e., pair of cues), then updated trial-by-trial using an auxiliary learning rate (free parameter) that measures how fast a subject learns about the skill of the Demonstrator. The full description of the model as well as the results of model comparison and modulation analysis were included in the Section "Modulation of imitation", **page 10**, **Fig 6**. The figure is also reported below as **Fig F12**.

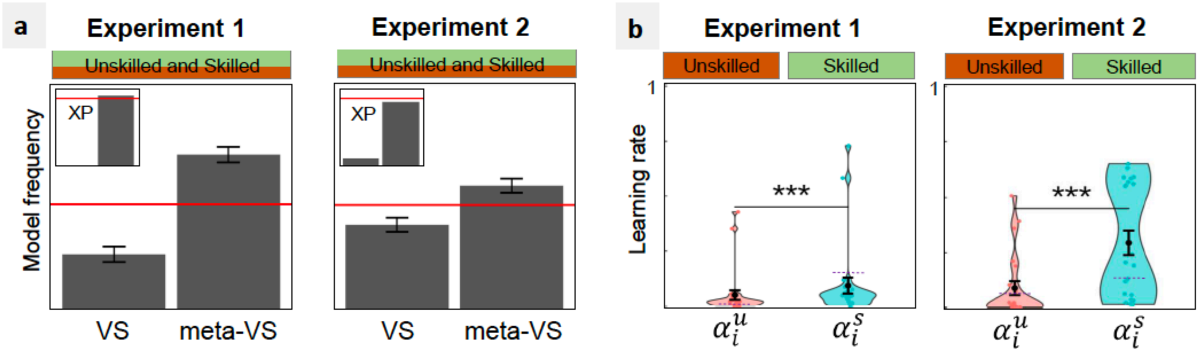

Fig F12. Meta-learning model. (**a**) Model comparison between the standard value-shaping model (VS) and value-shaping with meta-learning (meta-VS). 'Xp' denotes exceedance probability (**b**) The analysis of the average imitation rate over the experiment replicates the modulation effect with respect to the performance of the demonstrator (skilled vs. unskilled). The black dots represent that average imitation rate found fitting two different parameters.

Model comparison showed that value-shaping with meta-learning explained the subject's choices better than the standard value-shaping model (based on the AIC, cf. **Fig F12a**). y comparing the average imitation rates over the skilled and the unskilled conditions (cf. **Fig**

F12b), we were also able to reproduce the observed significant modulation of the imitation rates.

R2.3: On a related note, I do not understand why the UD condition has 20% optimal demonstrator choices, given that this means that the correlation between demonstrator choices and reward would be the same in both the UD and SD conditions, but with different signs. This should be clarified. Ideally, the authors would confirm in an additional control experiment that the same conclusions hold if the demonstrators choices are uncorrelated with reward (50% optimal choices), or at minimum, use simulation to determine expectations.

The Reviewer is correct, we defined an unskilled Demonstrator as a demonstrator choosing most frequently the suboptimal option. We believe that, as we clarified to the subjects that the option values were the same for both the Learner and Demonstrator, the label "unskilled" applies. In addition, implementing a 50% optimal response Demonstrator would have confounded two factors: optimality and variance of the Demonstrator response (indeed a 50% Demonstrator would continuously switch between options), while a 20% correct Demonstrator is suboptimal and equally stable.

We included these additional arguments in the Methods (**page 14**):

> Third, we manipulated the Demonstrator's performance by implementing skilled (80% of optimal, i.e., reward maximizing, responses) and unskilled (20% of optimal responses) behaviour. This allowed us to assess whether imitation was adaptively modulated. We did not implement unskilled performance as 50% of optimal responses, as it would have confounded two factors: optimality and variability (indeed a 50% correct Demonstrator would switch options continuously, while a 20% correct Demonstrator is sub-optimal but as stable as the skilled demonstrator).

In addition, we note that our analyses of Vostroknutov's experiment data also address this issue, as the average correct response rate of the demonstrator ranged from 53% to 92% (Skilled demonstrations: 0.84±0.03, Unskilled demonstrations: 0.64±0.05; mean±s.e.m.). This suggests that our main results hold across different levels of skills. As mentioned above, these results replicate our findings and are now included in the revised manuscript.

R2.4: It is not completely clear whether the outcomes of the demonstrator was shown (which typically is the case in certain conditions in this experimental paradigm). I concluded that they were not, given the focus on imitation and model description. Nonetheless, this could be clarified.

Thanks for pointing out this apparent lack of clarity. We have now clarified this aspect of the design in the revised manuscript. The rationale for not including outcome was that we were focusing on pure imitation, and not vicarious reinforcement; moreover, the presence of vicarious reward override imitation (one would not need to consider the Demonstrator's choices if her/his outcome is visible) [Selbing et al., *Cognition,* 2014; Safra et al., *PLoS computational biology,* 2019].

Results (**page 4**):

The outcome of the Demonstrator was never shown as we were interested in pure imitation and not vicarious reinforcement.

Figure 1 Legend (**page 3**):

The Demonstrator's outcome was not shown and replaced by a question mark '?'.

Methods (**page 14**):

First, as we were interested in pure imitation (and not vicarious trial-and-error learning) we did not include observation of the Demonstrator's outcome.

R2.5: I find the description of the model fitting procedure confusing. Under the sub header "Model comparison", its stated that models were fit using standard maximum likelihood, while under sub header "Parameter optimization", its stated that a Bayesian technique (LPP) is used. I do not follow how model estimation and parameter estimation are two different things, given that the parameter values are estimated in the maximum likelihood fit of the model to the data. This should be clarified.

The Reviewer here points out a lack of clarity concerning the model selection procedures, (which was also pointed out by Reviewer 1; see **R1.6** for further details about modifications made to improve clarity).

Parameter estimation was made twice at the subject level. First by maximizing log likelihood. Second, by maximizing the posterior probability of the data given the model and the parameters. Model selection was made at the group level by maximizing the posterior probability of the models using subject-specific AIC scores, which were itself derived from the maximum likelihood. On the other side, to analyze and compare the model parameters, we relied on parameters estimated maximizing the posterior probability.

We refer to **R1.6** for more details, but in short:

- Results using maximum LL estimated model parameters are the same as maximum LPP-estimated model parameters (see **Fig F9**), meaning that all the reported significant differences (between the private reward learning rate and the imitation learning rate and between the skilled and unskilled imitation learning rates) hold.

- The AIC gave an almost perfect model recovery, meaning that in synthetic data generated using known models, our model-fitting procedure indicates the true generative model as the 'winning' model.

R2.6: The authors repeatedly state that privately generated outcomes are over weighed compared to social information ("egocentric bias"). It is unclear how this conclusion is reached, which should be clarified throughout. Furthermore, little theoretical basis for the importance of an egocentric bias is provided.

The Reviewer raised a fair point (which was also raised by Reviewer 1; see **R1.5**). We conceived the concerned paragraph of the discussion not as establishing a claim, but rather as a possible interpretation of the findings, to be addressed by further research. We amended the introduction and the results section and downtoned the discussion to clarify these points and take into account this alternative interpretation.

Introduction (**page 3-4**):

> We also analyzed the parameters of the best winning model to determine whether or not, in the context of reinforcement learning, imitation more weight is given to information derived from oneself compared to the other [13]. The comparison of the private reward learning rate with the imitation learning rate was overall consistent with more weight given to privately generated information.

Results (**page 7**):

> We first assessed whether subjects put more weight on their freely obtained outcomes or on the choices of the Demonstrator. We found the private reward learning rates were significantly larger compared to the imitation learning rates (Fig 3.b). This was true in Exp 1 (Wilcoxon signed-rank test: V=300, p=1.192e-07). In Exp 2, the difference between private and imitation learning rates was even more pronounced when confronted with an Unskilled Demonstrator (Wilcoxon signed-rank test: V=251, p=1.431e-06), and still detectable when facing a Skilled Demonstrator (Wilcoxon signed-rank test: V=200, p=0.01558).

Discussion (**page 12**):

> This difference could derive from the fact that the Demonstrator's action, as a proxy of reward, implies an additional degree of uncertainty (one will never know whether the Demonstrator obtained a reward, after all). This difference could also derive from an egocentric bias, where self-generated information is systematically over-weighted.

R2.7: The authors should plot average trial by trial data, together with generative model predictions. In the current presentation, the reader cannot know how well the preferred model fits the data.

The reviewer here makes a good suggestion. To evaluate to what extent the winning model captures the observed behaviour, we now present in the supplementary materials the correlation between the actual and the simulated correct choice rate across trials. In all conditions the model account the behaviour very well (with intercept close to zero , slopes close to one and very reliable and significant correlations; see **Fig F13** below):

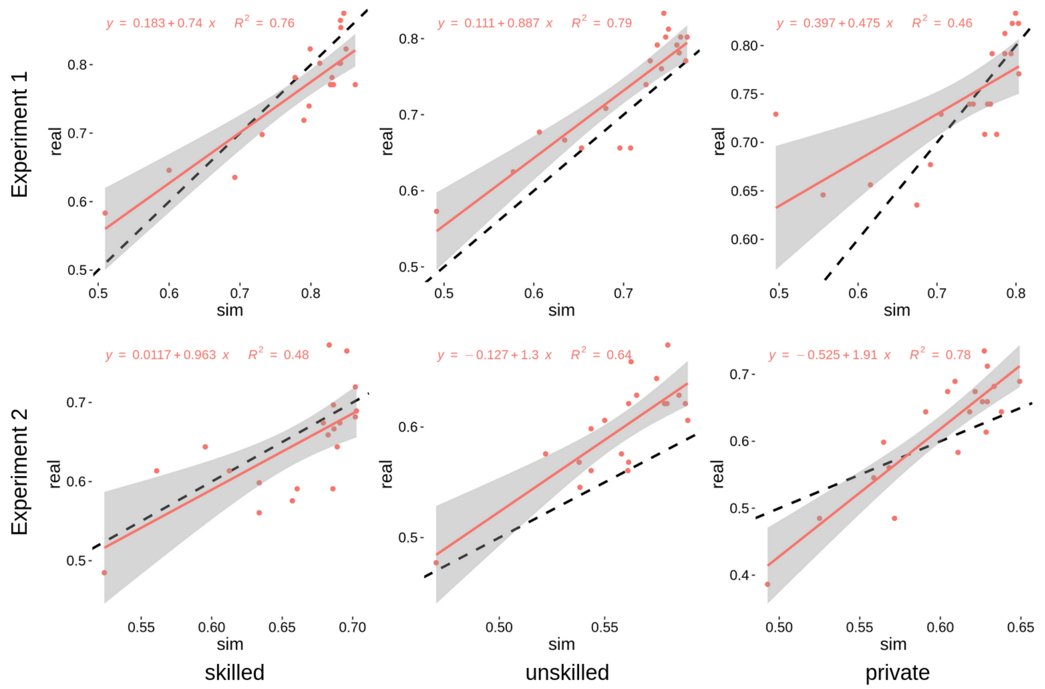

Fig F13. Across-trial correlation between the observed and model-predicted choices.

To further address the adequacy of our model, we also explored the correlation between predicted and simulated correct choice rate between-subject. Again, the results show that our model could correctly explain the observed behaviour also across subjects with intercept close to zero, slopes close to one and very reliable and significant correlations):

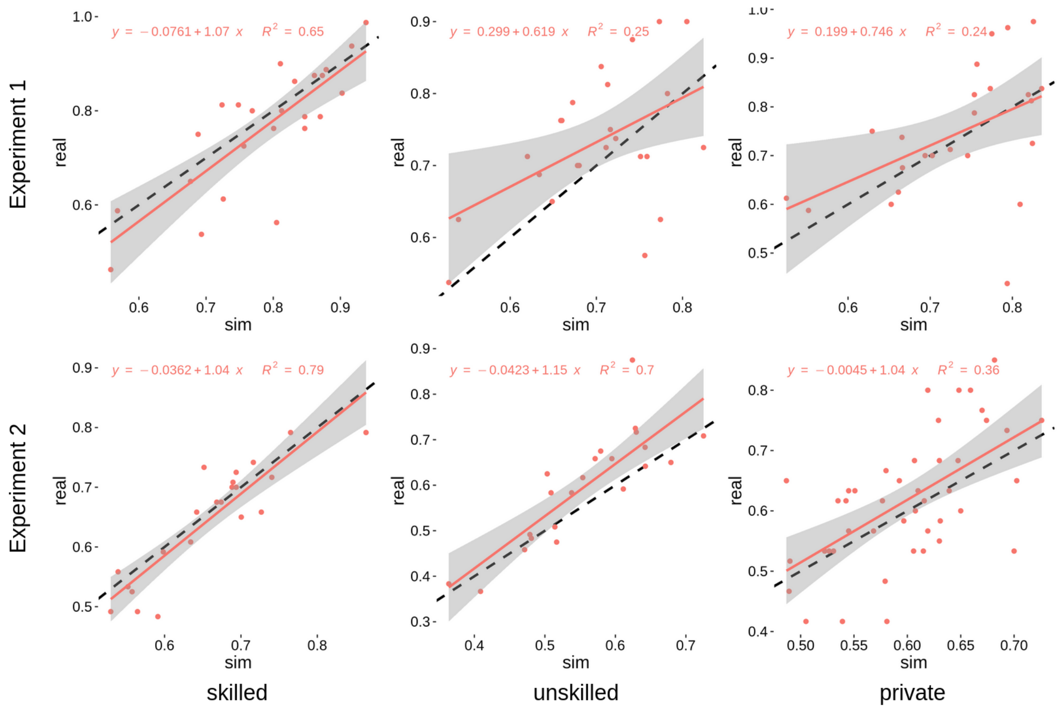

Fig F14. Across-subject correlation between the observed and model-predicted choices.

We mentioned these results in the revised manuscript and we present the Figures in the supplementary materials.

Results (**page 7**):

> Finally, in addition to analysing these distinctive features of our model given our design, we also checked whether our winning model was capable of capturing the observed behaviour in an unbiased manner. We found that the VS model captured well both between-trial and between-subject variance, as all the correlations displayed slopes very close to one, intercepts very close to zero (see Supplementary Fig S7 and Fig S8).

R2.8: It would be informative to include generative model simulations of the various hypotheses to determine the adaptive value of each postulated mechanism. This would put the various hypotheses on firmer theoretical ground.

We thank the reviewer for raising this question about model optimality that we find interesting. As suggested, we generated model simulations using prior distributions over parameters, using the same parameter values for the three imitation models. We then looked at the correct response rate in the imitation condition for each model (see **Fig F15**). We found that with skilled demonstrators, the model-based imitation model (MB) was the most optimal, whereas with unskilled demonstrators, the decision-biasing model (DB) was the most optimal. Interestingly, in both cases, the value-shaping model (VS) had an average performance, thus constituting a good compromise between model-based and decision biasing. We think that a proper exploration of the optimality would require a larger exploration of the task and parameters space. However, as Plos Biology published the Rebuttal letter, we are happy to show the findings here.

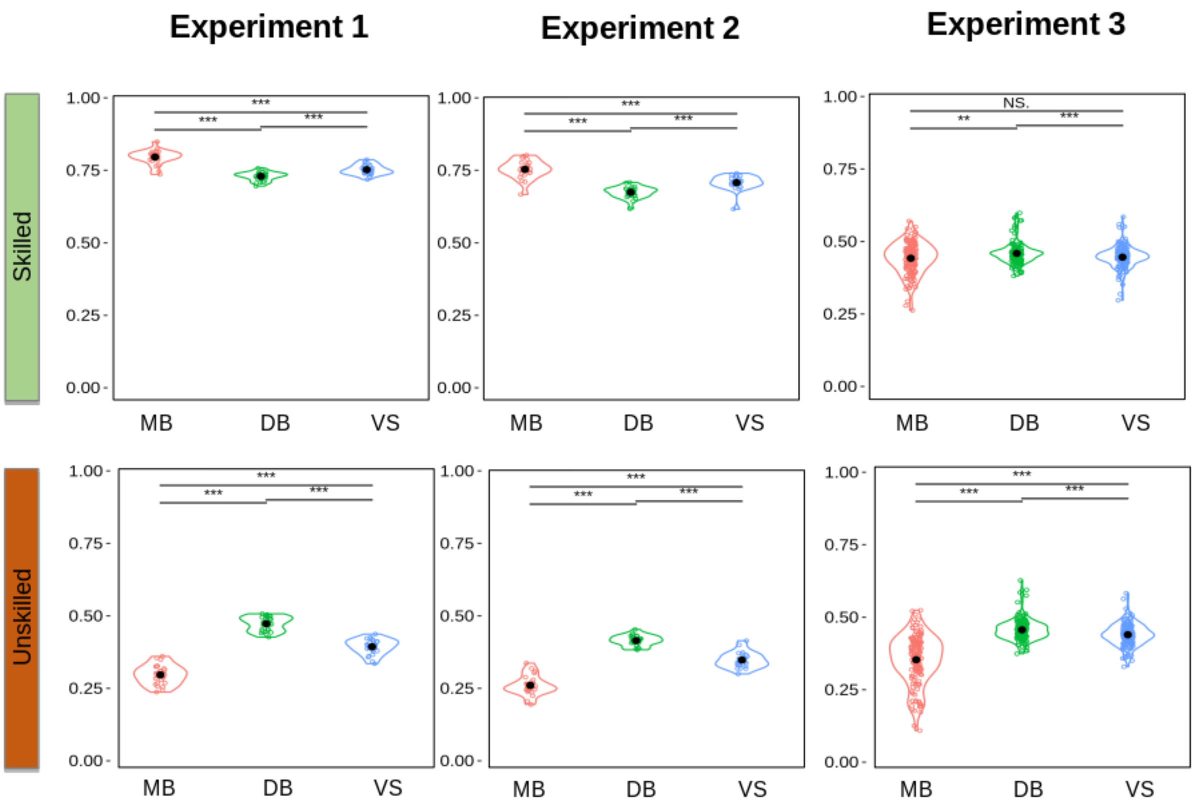

Fig F15. Model optimality comparison: simulated correct choice rate for each imitation model. Each data point is an average over 1000+ simulations per subject. For each experiment, we run N simulations per subject and per model (N=60 for Exp 1, N=30 for Exp 2 and N= 4 for Exp 3) to obtain around 1000 simulations per model. The same parameter values were used for the three models using the prior distributions. With a skilled demonstrator, the model-based imitation model (MB) is the most optimal, whereas with an unskilled demonstrator, the decision-biasing model (DB) is the most optimal. Interestingly, in both cases, the value-shaping model (VS) has an average performance, thus constituting a good compromise between model-based and decision biasing. Pairwise comparisons were made using paired t-tests (** p<0.01, *** p< 0.001).

R2.9: **I understood that the experiments varied the number of demonstrator choices shown before the individual choice. However, I could not find any information on how this was implemented (e.g., how often, how many), and if the varying amount of social information differentially affected behavior.**

The Reviewer is correct. As opposed to the standard Burke et al. (*PNAS*, 2010) design, we randomly varied the number of consecutive demonstrations, while keeping the constraint of having the same number of total demonstrations and private trials for each pair of stimuli (block). Over both experiments, the number of consecutive demonstrations varied between one and seven, with the additional constraint of not having more than three back-to-back demonstrations of the same action. We included this information in the revised version of the manuscript, in the “Methods” section (**page 14**):

> Second, to assess both the accumulation and the propagation of the imitation's influence, predicted by the value-shaped, but not by the decision-biasing model. In fact, over both experiments, the number of demonstrations in a row varied between one and seven demonstrations, with the additional constraint of not having more than three consecutive demonstrations of the same option.

We note that this feature was not shared by the newly included Experiment 3, where the maximum number of consecutive demonstrations was one, with sometimes more than one private trial (up to 16 private trials).

Reviewer #3:

R3.0: **This paper applies reinforcement learning models to the data of two experiments to investigate how imitation influences decision making. The results reveal that this influence can be best understood as "value shaping", meaning that observers change their valuation of a choice after seeing someone else choose it. By doing so, the current paper provides insight into the mechanisms underlying social learning.**

Before starting my review, I would like to note that I am not a modeler. Therefore, although I am familiar with reinforcement learning models and understand the authors' approach and results, I cannot comment on the specific implementation of the models.

I think this is an interesting paper with potentially important implications for how we understand social learning, and in particular for understanding why social influences can sometimes be very pervasive. That said, I also think the authors' operationalization of imitation has important restrictions and disagree with their operationalization of demonstrator skill. This is important, as it is not clear at this point to what extent these operationalizations influence the modeling results. Solving these issues will, in my opinion, require additional experiments. Please find a detailed overview of my comments below.

We thank the Reviewer for the positive evaluation and the constructive comments

Major comments

R3.1: In the presented experiments, imitation takes the form of seeing the output of someone's actions (i.e., their choice) and then deciding to copy that choice. One problem with this approach is that observers don't actually see the demonstrator's actions. That is, we know from research on automatic imitation that humans tend to imitate other people's actions and that this tendency relies on motor contagion (Heyes, 2011, Psych Bull). Presumably, in the RL models used here, motor contagion would take the form of "decision biasing". I therefore wonder if different models would be preferred if observers saw not only the outcome of the demonstrator's actions but also the actions themselves. Perhaps, in this case, a hybrid model combining decision biasing and value shaping would best explain the results? Addressing this issue will require either additional experiments or a careful discussion of the limitations of the current approach.

We thank the reviewer for raising this point. It is indeed correct that in our framework imitation operates in the *decision* space, rather than the *action* space. We opted for this implementation because we wanted to stay as close as possible to original - and widely used - design (Burke et al. PNAS, 2010). We also opted for this implementation because we wanted to avoid implicit communication via body signals and movement kinematics and wanted to focus on pure choice-based imitation (please note that an action observation protocol would also involve choice-

observation). Having acknowledged that, it is indeed true that this experimental choice leaves unanswered whether the same results would hold in a situation where the Demonstrator's motoric actions are observable. In this context, one hypothesis, suggested by the reviewer, is that imitation would switch to decision-biasing. An alternative hypothesis is that imitation would remain a value shaping process, but its intensity would be increased. In fact, by addition of the motor contagion process, the imitation learning rate could be as high as the private learning rate in this configuration. This would be consistent with a neural model linking action observation and brain valuation system (Lebreton et al. JNeurosci, 2011). Following the Reviewer's suggestion, we augmented the discussion of our paper with a paragraph that presents this limitation and mentions both hypotheses concerning what would be the consequence of action observation (We should note here that we assume that by referring to "the outcome of the demonstrator's action" the reviewer is referring to the demonstrator's *decision*. As we have clarified in the manuscript, choice outcomes were not presented to the participants to avoid vicarious learning).

Discussion (**page 12**):

> In our experiments, the actual motor responses (i.e., the action) necessary to implement the decision of the Demonstrator were not shown. We communicated them in abstract terms. We opted for this implementation for two reasons: we wanted to stay as close as possible to original - and widely used - design [6]; also we wanted to avoid implicit communication via body signals and movements and wanted to focus on pure imitation in the choice space (please note that an action observation protocol would also involve choice-observation). However, our experiments leave unanswered whether the same results would hold in a situation where the Demonstrator's actions are observable. Concerning imitation in an action-observation context, we recognize two possible scenarios that will be addressed by future studies. In one scenario, Value-shaping requires processing the Demonstrator's behaviour in the choice-space and therefore imitation would revert to a decision-biasing process prompted by a motor contagion process [39]. In another scenario, imitation recycles the same value-shaping computations in both the action- and the choice-space. Therefore, as the quality and quantity of social signal increases in the action-observation configuration, one could predict that the imitation learning rate could be as high as the private learning rate in these contexts (thus reverting the alleged egocentric bias).

R3.2: The manipulation of demonstrator skill is not a manipulation of skill. In the experiments, the "unskilled" demonstrator chooses the "suboptimal" option in 80% of the trials. A true unskilled demonstrator, on the other hand, would pick the suboptimal option in 50% of the trials. The fact that the "unskilled" demonstrator chooses the suboptimal choice more often implies that this demonstrator has different preferences (i.e., for some reason they prefer to get less reward). This is not trivial: a true unskilled demonstrator is a demonstrator that contains no information and should therefore be ignored to optimize reward. An unskilled demonstrator as implemented here, on the other hand, is a demonstrator that should be counter-imitated to optimize reward. It is currently unclear to what extent this can explain the modeling results: is the value shaping mechanism a general mechanism or is it a mechanism that only applies to this specific situation? To address this question, I think additional experiments are needed that test whether the behavioral results differ depending on the operationalization of "skill" and whether the model is able to capture this.

We thank the Reviewer for this suggestion (also shared by Reviewer 2; see **R2.3**). The Reviewer is correct, we defined an unskilled Demonstrator as a demonstrator choosing the suboptimal option more frequently. We believe that, as we clarified to the subjects that the option values were the same for both the Learner and Demonstrator, the label “unskilled” applies. In other words, we emphasized that the demonstrator does not intend to deceive the participant. Importantly, implementing a 50% optimal response Demonstrator would have confounded two factors: optimality and variability of the Demonstrator response (indeed a 50% Demonstrator would continuously switch between options), while a 20% correct Demonstrator would be suboptimal but equally stable as the Skilled Demonstrator.

We included these additional arguments in the Methods (**page 14**):

> Third, we manipulated the Demonstrator’s performance by implementing skilled (80% of optimal, i.e., reward maximizing, responses) and unskilled (20% of optimal responses) behaviour. This allowed us to assess whether imitation was adaptively modulated. We did not implement unskilled performance as 50% of optimal responses, as it would have confounded two factors: optimality and variability (indeed a 50% correct Demonstrator would switch options continuously, while a a 20% correct Demonstrator is sub-optimal but as stable as the skilled demonstrator).

In addition, to further address this issue we included a new experiment (N=302) where demonstrator skills ranged from 53% to 92% (Skilled demonstrators: 0.84±0.03, Unskilled demonstrators: 0.64±0.05; mean±s.e.m.). As mentioned above, the analyses of this new dataset replicated our findings and are now included in the revised manuscript (see **Figure F16**; see also **R2.1** & **R2.3**). We included the new experiment in the revised manuscript in a dedicated result section.

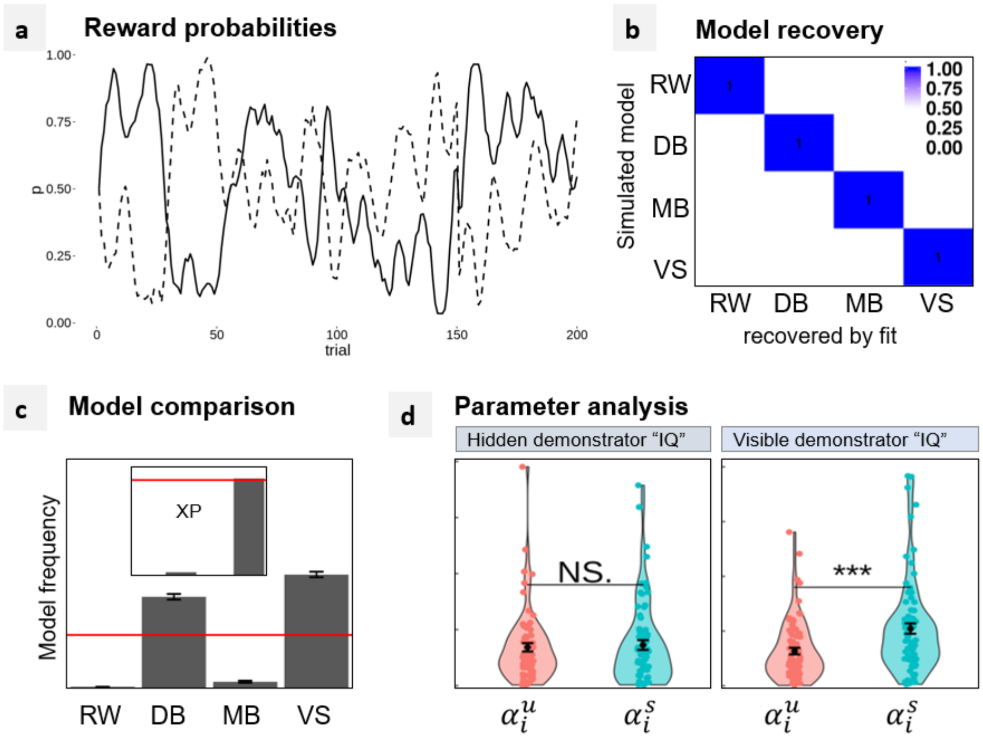

Fig F16. Replication of the main results on a third dataset (N=302). (a) Reward contingencies over trials for the two options (bold and dotted line, respectively) changed following a random walk. (b) Model recovery of the four main computational models using the AIC on simulated data using Experiment 3 specifications. The numbers written in the matrix indicate the exceedance probability, the intensity of the color indicates the posterior probability. (c) Model comparison. The value-shaping model VS is the winning model. (d) Parameter analysis: comparison of the imitation rate between subjects exposed to skilled and unskilled demonstrators. Leftmost panel: group for which the IQ of the demonstrator was not disclosed; rightmost panel: group for which the IQ of the demonstrator was disclosed.

R3.3: Related to my first point, is it possible that hybrid models (e.g., a model combining model-based imitation and value shaping) would explain the results even better than a model with value shaping alone? Please discuss.

The implementation suggested by the Reviewer 3 is, in principle, possible. However, we excluded it because we believe that it is conceptually problematic. Specifically, implementing both mechanisms, i.e. using observations both to build a *separate* model of the demonstrator to then modify the subject's own value function, would be redundant and would defeat the purpose of the modelling exercise by conflating the two mechanisms. However, as the Reviewer raised the point, we implemented two versions of the proposed model, one using the same parameter for both value-shaping and model-based imitation, and another with different parameters for each mechanism. We compared these two implementations to both standard model-based imitation and value-shaping. Model comparison results show that the first implementation (HYB1) performs better than the second (HYB2). However, our value-shaping model outperforms both hybrid models in all the three experiments (cf. **Fig F17** below).

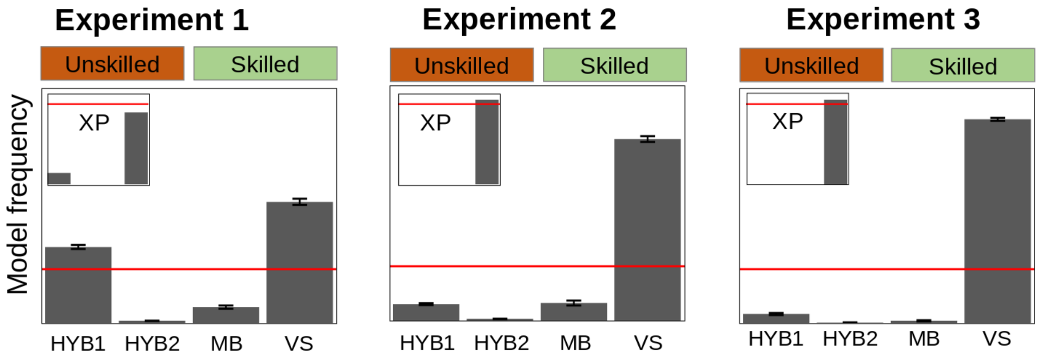

Fig F17. Model comparison between model-based imitation (MB), value-shaping (VS), and two implementations of a hybrid model, HYB1 and HYB2 across three experiments.

R3.4: P. 14: "We selected the AIC as a metric after comparing its performance in model recovery along with other metrics such as the BIC (see the paragraph about model recovery)". Why was the AIC chosen over the BIC? The AIC is known to sometimes overfit. Do the results change if the BIC is used instead? Also, I could not find anything on the BIC in the model recovery paragraph in contrast to what is suggested in the cited sentence. Please fix or clarify.

The Reviewer is correct, in general, the AIC is less stringent than the BIC. The problem is the BIC is often overpenalyzing. However, in our case the AIC gave a very good model recovery (meaning that we were capable of retrieving the correct model in simulated datasets where the ground truth is known). This is why we safely included the AIC results. The model recovery results for Experiment 1 and 2 where already presented in first version of the manuscript and we report them here in **Fig F18**:

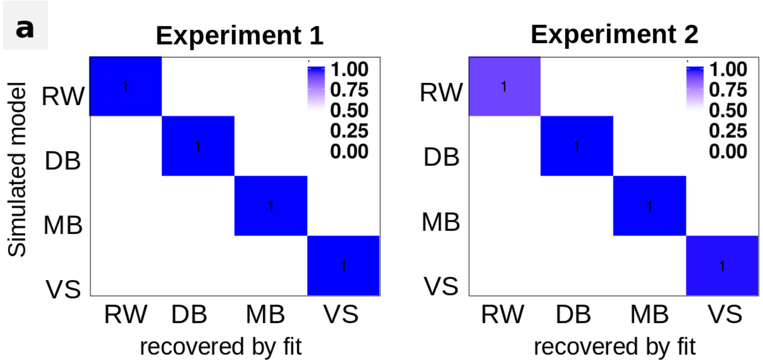

Fig F18: Model recovery using the AIC in Experiment 1 and 2. See **Fig F16** above for the same result in Experiment 3. The number written on the matrix indicates the exceeded probability, the intensity of the color indicates the model frequency.

We also note that, except from rejecting the RW, as the three main imitation models present the same number of free parameters they can be compared just using the maximum likelihood. In other words, the parameters penalization only concerns the comparison between the RW and

all the other models of imitation. So, once we had excluded the RW model (at least for skilled Demonstrators), the other models (DB, MB, and VS) could have been compared just using the maximum likelihood without incurring any danger of overfitting. Here we report the model comparison results based on the likelihood for the three experiments, restricting ourselves to the three imitation models (see **Fig F19**). Overall, the results confirm our conclusions (even more strongly in Experiment 2 and Experiment 3 that present the bigger sample sizes). The VS model outperforms DB and MB.

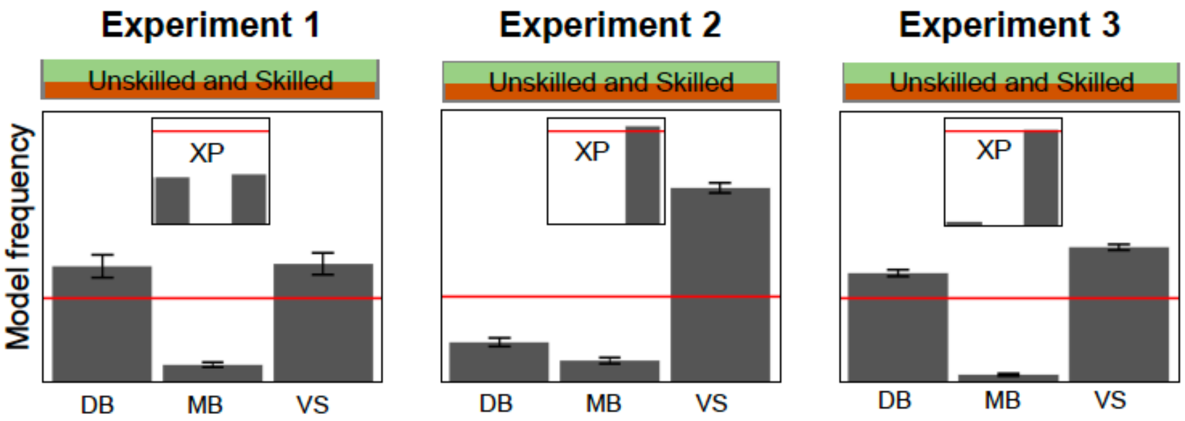

Fig F19: model comparison using the likelihood as approximation of the model evidence and restricting the comparison to the three imitation models. Note that the models have identical numbers of free parameters.

We mention this result in the revised version of the manuscript and included this figure as a Supplementary **Figure S10**.

Results (**page 6**):

> We also note that in our model space, parameters penalization only concerns the comparison between the RW versus the all imitation models. As the three imitation models have the same number of free parameters, the DB, MB, and VS models can be compared just using the maximum likelihood without incurring over-fitting. Overall, model comparison results (based on the maximum likelihood comparison restricted to the DB, MB, and VS models) confirm our conclusions that the VS outperforms DB and MB (see Supplementary Fig S9).

Finally, beyond relative model comparison using approximation of model evidence, following Reviewer 1’s suggestion, we included in the manuscript a qualitative analysis of specific behavioural signature of the imitation models (accumulation and propagation of imitation), which further support our model comparison conclusions (see **R1.1** for more details).

Minor comments

R3.5: Did the authors check whether participants believed they were actually playing against someone else? In my experience, participants tend to be skeptical of such cover stories.

In our experiments, we did not systematically check whether subjects believed in the cover story, as we were afraid to prompt a negative response. On the other hand,. we put a strong effort to make the cover story as credible as possible, by having a pair of subjects coming exactly at the same time, taking breaks also at the same time etc. We also asked each participant to send a photo in advance in order to personalize the avatar presentation in the behavioral task. Finally, we note that in Experiment 3, following behavioural economics standards, involved no deception and all the results hold.

R3.6: P 7.: "Over two experiments, we found that whenever imitation is adaptive (i.e., Skilled Demonstrator), it takes the computational form of value shaping, which implies that the 185 choices of the Demonstrator affect the value function of the Learner." --> Presumably, value shaping is always happening, but can be top-down modulated to have more or less effect on behavior. This is also how I interpret the "Adaptive modulation of imitation" section in the discussion. The cited sentence, on the other hand, makes it appear as if value shaping only happens for skilled demonstrators. Please change or clarify.

Thanks for spotting this, we clarified the sentence as follows (Discussion, **page 9**):

> Over three experiments, featuring different outcome contingencies and different ratios between private and observational learning trials, we found that imitation takes the computational form of value shaping, which implies that the choices of the *Demonstrator* affect the value function of the Learner . On top of that, we found that imitation is modulated by a meta-learning process, such that it occurs when it is adaptive (i.e. *Skilled Demonstrator*)

We also note that one of the major revisions of the paper consisted in adding a meta-learning model that precisely addresses the process of modulating imitation as a function of the Demonstrator's performance (see also **R1.2** and **R2.2**)
