# Supplementary material for: The actions of others act as a pseudo-reward to drive imitation in the context of social reinforcement learning
Source: PLoS Biol. 2020 Dec 8;18(12):e3001028. doi: 10.1371/journal.pbio.3001028 (PMC7723279; doi:10.1371/journal.pbio.3001028)
Supplement: Attachment — Submitted filename: Najar_PlosBio_Rebuttal2.docx [file pbio.3001028.s013.docx]

**Point 1**

However, we would like you to consider changing your title to make it more accesible to a broad readerships and to convey the central biological message. We offer you this suggestion, but would be happy to discuss alternatives:

"The actions of others act as a vicarious reward in the context of reinforcement learning."

Concerning your title, we appreciate the suggestion and the need to make the title as accessible as possible to a broad audience (and we agree that the term “model-free” is very technical). However, concerning your specific proposition, we are worried as, in this context, the term vicarious is traditionally used to describe a process that we did not investigate (precisely observing the outcomes obtained by another person).

Please find below a counterproposition:

“Computational mechanisms of imitation in the context of reinforcement learning”

**Point 2**

Please also check if the word "imitation" was not mistakenly inserted in the following sentence in the Introduction: “We also analyzed the parameters of the best winning model to determine whether or not, in the context of reinforcement learning, imitation more weight is given to information derived from oneself compared to the other [13].”

What we meant with this sentence is “whether information derived from oneself is weighted more (receives more weight) compared to information derived from others”. We therefore do not believe that the work imitation should be there. However, thanks for spotting that the sentence was not clear. It could be changed as follows:

“We also analyzed the parameters of the best winning model to determine whether in the context of reinforcement learning more weight is given to information derived from oneself compared to information derived from others [13].”

Can you confirm that this makes more sense to you? Also please note that in our documents reference [14] (not reference [13]) follows this sentence.

**Point 3**

Also consider changing "her" for "their" in the following sentence in the Abstract: "The first hypothesis, decision biasing, postulates that imitation consists in transiently biasing the learner's action selection without affecting HER value function"

We do not have any issue per se in changing “her” for “their” in the abstract. However, please note that we used the feminine gender in several other instances in the manuscript when referring generically to the Learner of the Demonstrator. I am ok for you to change all occurrences, but I am afraid it would generate some additional work and some occurrence may still be missed.

**Point 4: *Published Peer Review History***

Please note that you may have the opportunity to make the peer review history publicly available. The record will include editor decision letters (with reviews) and your responses to reviewer comments. If eligible, we will contact you to opt in or out. Please see here for more details:

<https://blogs.plos.org/plos/2019/05/plos-journals-now-open-for-published-peer-review/>

I am ok to publish the peer review history

**Point 5: *Early Version***

Please note that an uncorrected proof of your manuscript will be published online ahead of the final version, unless you opted out when submitting your manuscript. If, for any reason, you do not want an earlier version of your manuscript published online, uncheck the box. Should you, your institution's press office or the journal office choose to press release your paper, you will automatically be opted out of early publication. We ask that you notify us as soon as possible if you or your institution is planning to press release the article.

We have no problem in publishing a early version online, but we would like to be able to coordinate with our press office an exact embargo date, in order to coordinate the press release.

**Point 6: ETHICS STATEMENT:**

-- Please indicate within your manuscript if you experimental protocol was approved by your Institutional Review Board or a similar ethical committee.

-- Please include the ID number of the approved protocol.

Thanks for spotting this, we have added the following statement:

"The study was approved by the local Institutional Review Board (C15-98 / 58-17

**Point 7: DATA POLICY:**

-- Please edit your README file in <https://github.com/hrl-team/mfree_imitation> to explain how your data was analyzed to generate the plots displayed in Figures Fig 3ab, 4, 5acd, 6ab, S1, S2, S3abc, S4, S5, S6., S7, S8, and S10.

-- In addition, please deposit spreadsheets with the individual numerical values that underlie the summary data displayed in the aforementioned graphs. The numerical data provided should include all replicates AND the way in which the plotted mean and errors were derived (it should not present only the mean/average values). For an example see here: <http://www.plosbiology.org/article/info%3Adoi%2F10.1371%2Fjournal.pbio.1001908#s5>

-- Please also ensure that figure legends in your manuscript include information on where the underlying data can be found, and ensure your supplemental data file/s has a legend.

-- Please ensure that your Data Statement in the submission system accurately describes where your data can be found.

Thanks for spotting this, we improved the online data in order to make them more readable.

**Point 8**

Please confirm that you either created the following figure panels, or that you will replace them with an open source alternative: illustrations/graphics in Fig 1 (panel B)

We took the avatar image and modified it from svg repo under CC0 License (No copyright) : https://www.svgrepo.com/svg/43652/avatar

Licence: https://www.svgrepo.com/page/licensing
